# Determination of the p*K*_aH_ of Established Isothiourea Catalysts

**DOI:** 10.1002/ejoc.202401412

**Published:** 2025-01-28

**Authors:** Lukas S. Vogl, Matthias Bechmann, Mario Waser

**Affiliations:** [a] Institute of Organic Chemistry, Johannes Kepler University Linz, Altenbergerstrasse 69, 4040 Linz, Austria

**Keywords:** Brønsted bases, Isothioureas, p*K*_a_, NMR

## Abstract

Isothioureas (ITUs) represent a powerful family of (chiral) Lewis base organocatalysts. Interestingly, the Brønsted basicity of these frequently used compounds has so far not systematically been investigated. Thus, we have now determined the p*K*_aH_ values of the most privileged (chiral) ITUs in acetonitrile (ACN) and DMSO by using NMR. Employing Wallace’s chemical shift imaging NMR method, the herein investigated ITUs were found to be weak Brønsted bases with p*K*_aH_ values in the range of 16.8–17.9 in ACN and 6.3–7.8 in DMSO.

## Introduction

**Scheme 1:**
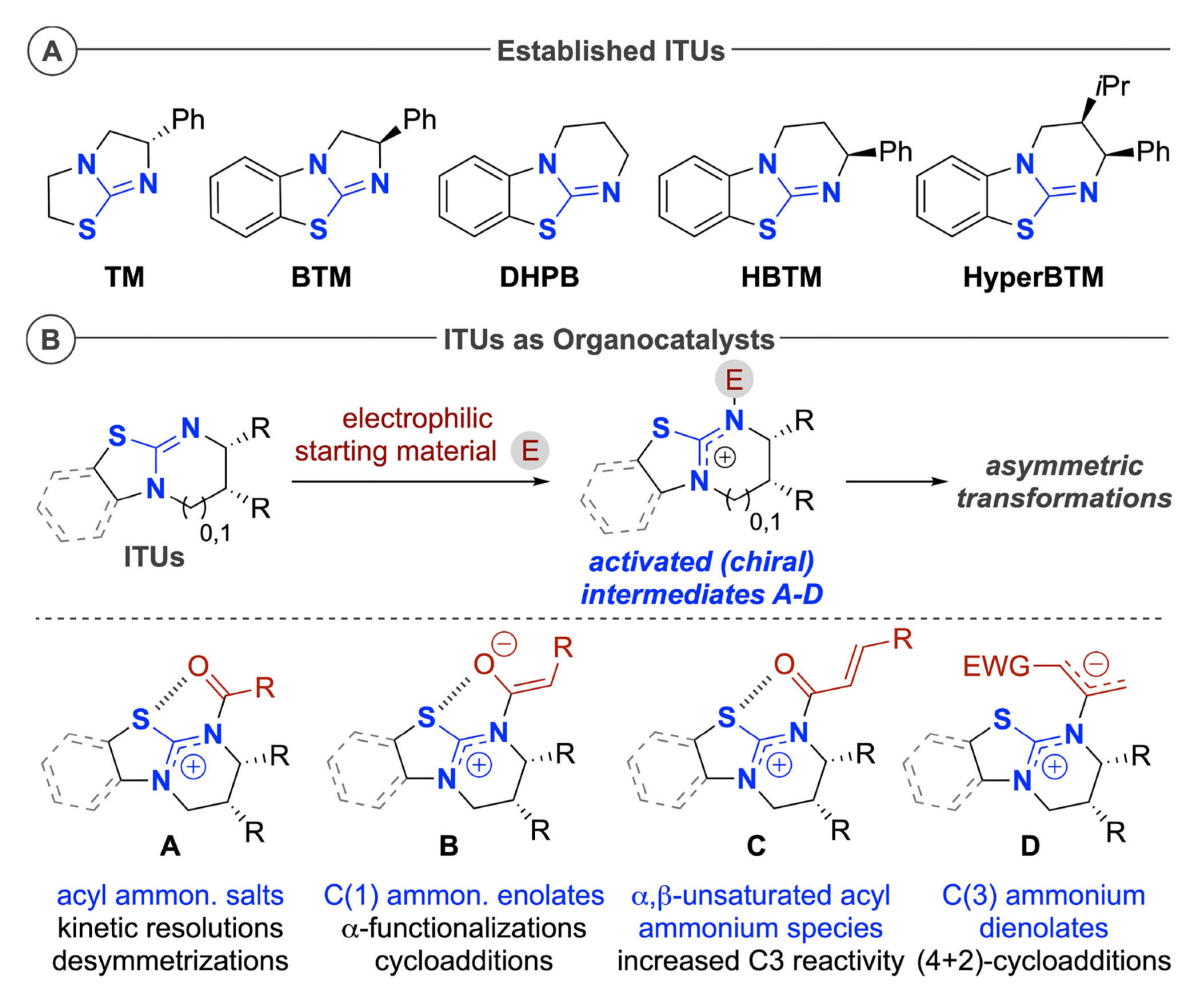
(A) Commonly used privileged isothioureas (ITUs) and (B) established activation modes and intermediates in ITU-catalyzed transformations.

Isothioureas (ITUs; Scheme 1A) have found widespread use as easily available and bench-stable (chiral) Lewis basic organocatalysts^[1]^ over the course of the last two decades. These catalysts usually act by adding to electrophilic starting materials via their nucleophilic N-group, forming highly activated chiral intermediates which can then undergo a multitude of different transformations (Scheme 1B).^[1–13]^ The seminal report that revealed the potential of ITUs for asymmetric catalysis came from Birman’s group in 2006, who successfully utilized the commercially available drug tetramisole (**TM**) and its benzannulated derivative benzotetramisole (**BTM**) as catalysts for asymmetric acyl-transfer reactions (via in situ formation of acyl ammonium salt intermediates **A**).^[2]^ Since then, a broad variety of highly efficient acyl-transfer reactions for (dynamic) kinetic resolutions and desymmetrization reactions have been reported.^[3–6]^ Analogously, ITUs have been well-established for asymmetric silylations of alcohol derivatives.^[7]^ Furthermore, ITUs have been very successfully utilized for: i) the generation and utilization of chiral C(1) ammonium enolates **B** (which can undergo asymmetric α-functionalizations and cycloadditions);^[8–10]^ ii) the formation of reactive chiral α,β-unsaturated acyl ammonium acceptors **C**;^[11,12]^ and iii) to access C(3) ammonium dienolates **D** which can undergo formal (4+2)-cycloadditions.^[13]^

Interestingly, while a lot of the crucial factors that have an impact on the catalytic potential of ITUs like their tendency to undergo well-defined intramolecular 1,5-O…S chalcogen bonding interactions in intermediates **A**-**C** (n_O_→σ*_C-S_),^[3b,14]^ as well as their nucleophilicity and Lewis basicity have been the subject of previous investigations,^[15]^ only little is known about the Brønsted basicity of these amidine-type structures. Surprisingly, only recently^[16]^ some experimentally determined p*K*_a_ values of the conjugated acids of selected achiral ITUs based on the BTM-scaffold were reported (here potentiometric titration revealed p*K*_aH_^[17]^ values around 8 in H_2_O/MeOH mixtures). To the best of our knowledge however, the p*K*_aH_ of the privileged (chiral) isothioureas depicted in Scheme 1A have so far not been systematically determined. For us, this lack of data came as a surprise, especially when we recently found that, depending on the application, these catalysts may also allow for the formation and utilization of C(1) ammonium enolates without the need for an additional external base.^[18]^ This observation made us wonder about the Brønsted basicity of these compounds in relation to other commonly used organic bases.

To gain a better understanding of the key properties of the most-commonly used (chiral) ITU derivatives (Scheme 1A), we have now focused on the experimental determination of their p*K*_aH_ values in the common organic solvents DMSO and acetonitrile (ACN). Having these fundamental thermodynamic data at hand thus allows for a direct comparison of these bases with other commonly used organic Brønsted bases.^[19,20]^

## Results and Discussion

**Figure 1:**
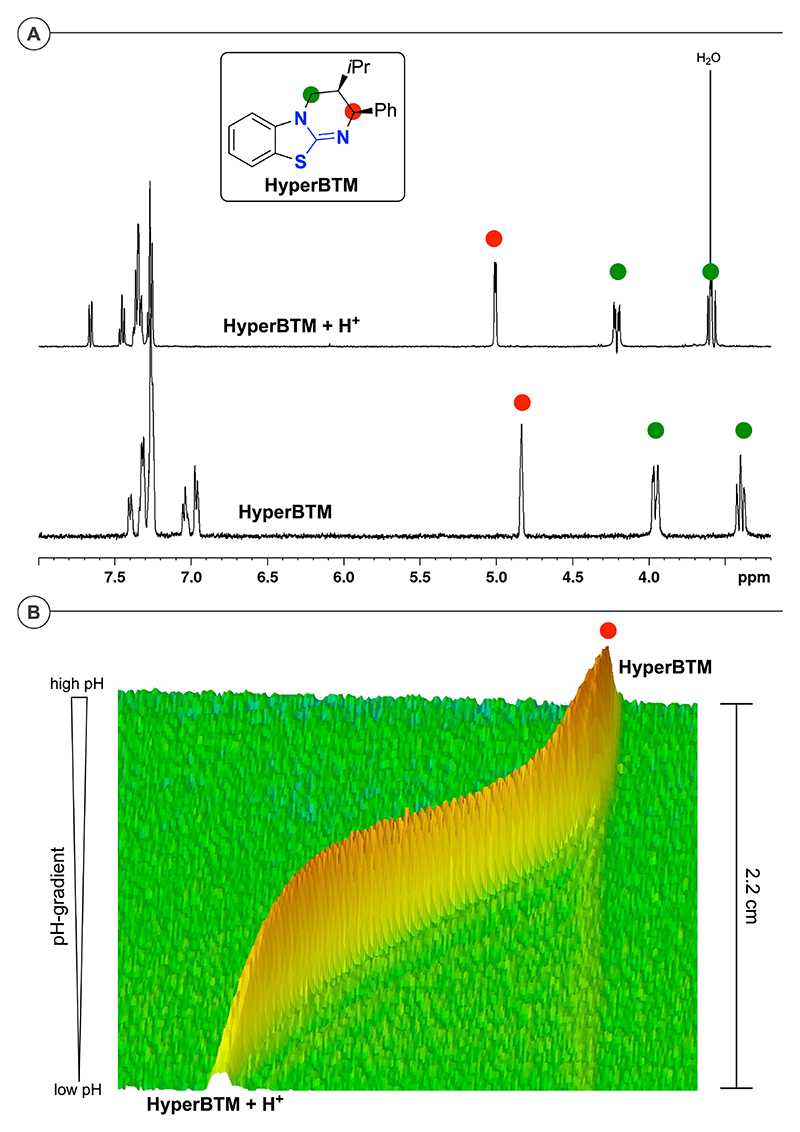
(A) Region of the ^1^H NMR spectra of **HyperBTM** in its free base form and in its protonated form that can be used for the determination of the p*K*_aH_ (recorded in non-deuterated DMSO with solvent suppression); (B) Chemical shift of the proton adjacent to the basic nitrogen in dependence of the position in an NMR tube with a pH gradient along the length of the tube.

To determine the p*K*_aH_ values in a comparable and systematic manner, we chose a chemical shift imaging NMR method that has recently been developed by Wallace and co-workers.^[21]^ This method allows for the determination of the p*K*_aH_ of organic bases in a straight-forward “one-shot” manner in different organic solvents and was found to be well-applicable to our ITUs. In a nutshell,^[21,22]^ this method works by analyzing the chemical shifts of the compound of interest in dependence of the pH. Based on the ratio between protonated species and free base at a certain pH the pKa of the conjugated acid can be determined (Figure 1). Avoiding numerous experiments at different pH values, Wallace’s method allows to carry this out in a single experiment by measuring samples of the base of interest (our ITU) with two basic reference compounds of known p*K*_aH_ in an NMR tube with a pH-gradient along the length of the tube (lowest pH on the bottom, highest pH on the top).^[21,22]^ By utilizing a gradient-equipped NMR spectrometer it is possible to record individual ^1^H NMR spectra of small “slices” of the sample (128 slices over a length of 2.2 cm), thus providing chemical shifts as a function of the position/location (Figure 1B). By aid of the two reference compounds it is possible to exactly assign the pH in each individual slice and once this is known one can then calculate the p*K*_aH_ from the ITU-H^+^/ITU ratio within each slice.^[21,22]^ To simplify analysis and interpretation of the spectra reference compounds that do not show any peak overlap with the relevant peaks of our targets were chosen.^[22]^

**Figure 2:**
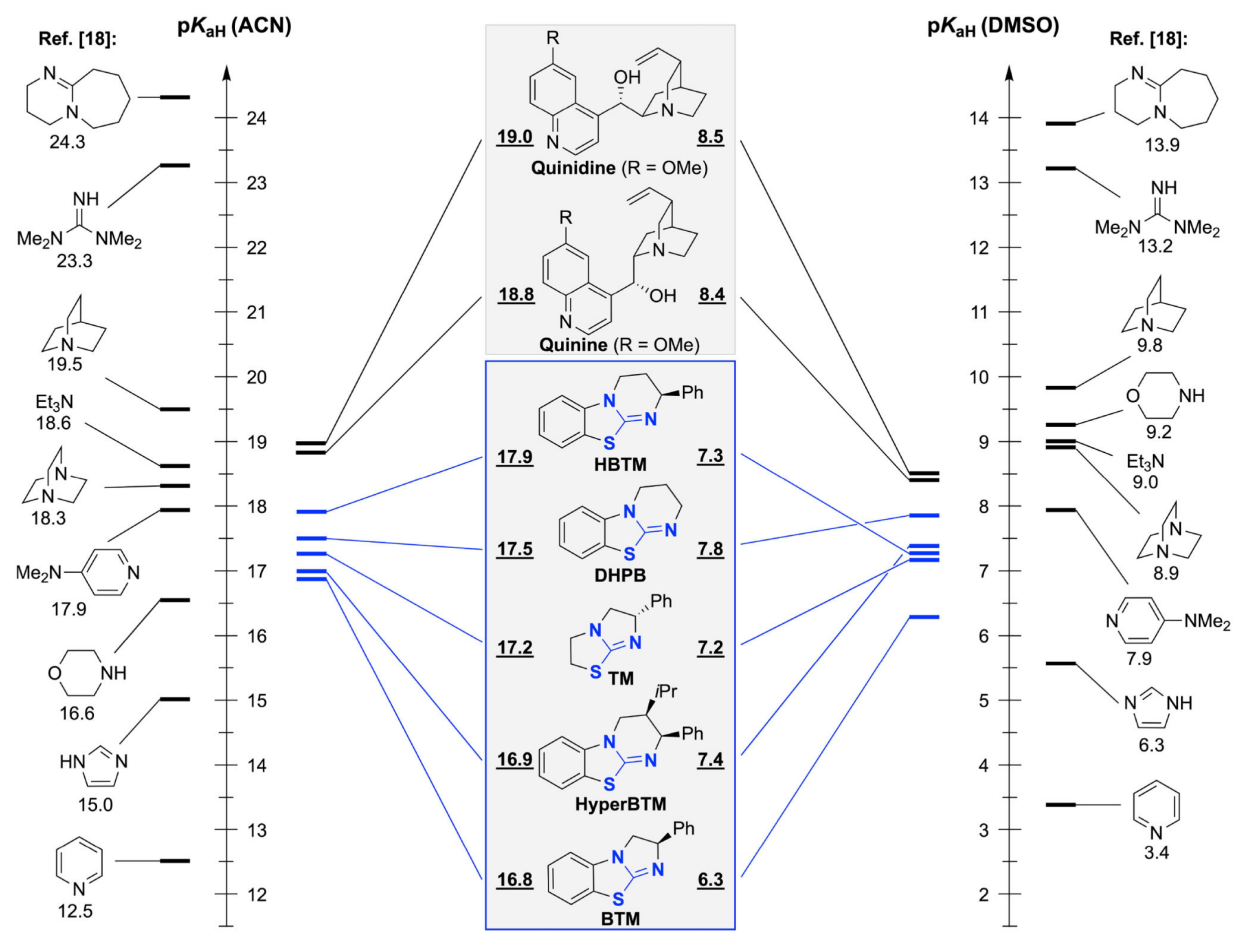
Experimentally determined p*K*_aH_ values of ITUs (and some Cinchona alkaloids) in ACN and DMSO in comparison to established organic Brønsted bases.^[19]^

Gratifyingly, this NMR methodology worked rather well for our ITUs of interest, thus allowing us to determine their p*K*_aH_ values in both, acetonitrile and DMSO. By looking at the obtained values and comparing them with established organic bases^[19]^ (Figure 2), it becomes obvious that ITUs are relatively moderate Brønsted bases only. With p*K*_aH_ values in the region of 16.8–17.9 in ACN and 6.3–7.8 in DMSO their most basic members show a similar basicity as DMAP. Interestingly, although all derivates possess rather similar p*K*_aH_ values spanning roughly one order of magnitude only, some note-worthy trends were observed. First of all, the ring size significantly effects the basicity, as illustrated by comparing the 5-ring-based **BTM** with its 6-ring homologue **HBTM**, which is one order of magnitude more basic in both solvents. Furthermore, it turns out that the benzannulation leads to a decrease in p*K*_aH_ as shown for **BTM** in comparison to **TM**. Interestingly the relative order of basicity somewhat depends on the solvent. This is especially pronounced for the 6-ring fused derivatives HBTM, HyperBTM and DHPB (in ACN: HBTM > DHPB > HyperBTM; in DMSO: DHPB > HyperBTM > HBTM).

With these p*K*_aH_ values for the established ITUs at hand, we also wanted to set them in relation to Cinchona alkaloids (i. e. the privileged derivatives quinine, quinidine, cinchonine, and cinchonidine). Those naturally occurring tertiary amines are frequently used in asymmetric (organo)-catalysis^[23]^ and their p*K*_aH_ values in H_2_O or aqueous solvent mixtures are known.^[19,24]^ Surprisingly however, we could not find a systematic study on their p*K*_aH_ values in ACN or DMSO. We thus determined them by NMR as well and found these Cinchona alkaloids being at least one order of magnitude more basic than our ITUs (Figure 2 gives the value for quinine and quinidine but we also determined them for cinchonidine and cinchonine which both have identical p*K*_aH_ values (18.4 in ACN; 8.2 in DMSO)).^[22]^

## Conclusions

Isothioureas (ITUs) emerged as powerful (asymmetric) Lewis base organocatalysts over the course of the last two decades. Surprisingly to us, the Brønsted basicity of these easily accessible amidine-type compounds has so far not systematically been investigated.^[16]^ We thus now determined the p*K*_aH_ values of some of the most commonly used (chiral) ITUs in acetonitrile and DMSO by using Wallace’s NMR methodology.^[21]^

Utilizing this straightforward approach, the herein investigated ITUs were found to be weak bases with p*K*_aH_ values in the range of 16.8–17.9 in ACN and 6.3–7.8 in DMSO. Furthermore, we also determined the p*K*_aH_ values for privileged Cinchona alkaloid derivatives, which are around one order of magnitude more basic than the investigated ITUs.

## Supplementary Material

SI

## Data Availability

The data that support the findings of this study are available in the supplementary material of this article.
