# Supplementary material for: Determination of the pKaH of Established Isothiourea Catalysts
Source: European J Org Chem. Author manuscript; Available in PMC 2025 Jul 5. (PMC7617867; doi:10.1002/ejoc.202401412)
Supplement: SI [file EMS206750-supplement-SI.pdf]

European Journal of Organic Chemistry

Supporting Information

Determination of the pK_{aH} of Established Isothiourea Catalysts

Lukas S. Vogl, Matthias Bechmann, and Mario Waser*

Table of Contents

1. General information 2
2. Datapoint selection 3
3. Sample preparation 4
4. Indicator and acid Data 5
5. Titration Data 6
    5.1. Acetonitrile 6
    5.2. DMSO 6
6. References 7
7. NMR Spectra: 8
    7.1. Acetonitrile 8
        7.1.1. Quinidine: 8
        7.1.2. Cinchonine: 9
        7.1.3. Cinchonidine: 10
        7.1.4. Quinine: 11
        7.1.5. N-Ethyldiethanolamine: 12
        7.1.6. Tetramisole: 13
        7.1.7. HBTM: 14
        7.1.8. DHBP: 15
        7.1.9. BTM: 16
        7.1.10. HyperBTM: 17
    7.2. DMSO: 18
        7.2.1. BTM: 18
        7.2.2. Cinchonine: 19
        7.2.3. Cinchonidine: 20
        7.2.4. Quinidine: 21
        7.2.5. Quinine: 22
        7.2.6. Tetramisole: 23
        7.2.7. HBTM: 24
        7.2.8. HyperBTM: 25
        7.2.9. DHBP 26

1. General information

All NMR experiments were recorded manually on a Bruker Avance DRX 500 MHz spectrometer with a broad band observe probe, which is the property of the Austro-Czech NMR-Research Center "RERI-uasb". The CSI experiments were carried out using the gradient phase encoding of *Wallace et al.* [1,2] The measurement parameters were based on the work of Wallace et al. [1,2] Water suppression was carried out using excitation sculping (Bruker Library zgesgp). [3] The phase encoding gradient pulse was 284 µs and varied from -61.5 to 61.5 $\frac{G}{cm}$ in 128 increments. For all CSI measurements, 16 dummy scans were run before acquisition, and 8 scans acquired for each of the 128 slices, with an acquisition time of 1 s and a relaxation delay of 0.5 s. After acquisition a spoil gradient of 30.8 $\frac{G}{cm}$ was included to destroy any remaining transverse magnetisation. The transformation of the time domain data files was carried out without zero filling in the indirect dimension but with zero filling in the direct dimension and sine bell apodisation. A total experiment took 28 minutes, and a theoretical spatial resolution of 0.17 mm was achieved.

The measurements were referenced relative to the internal standard tetramethyl silane (TMS: δ = 0 ppm for ^{1}H-NMR in both DMSO-h_6 and acetonitrile-h_3). The shimming was done on the respective ^{1}H solvent peak. All experiments were carried out in non-deuterated solvents and therefore no lock was possible. All CSI experiments were measured at 20 °C.

The solid acids, the pK_a standards and purification agents were purchased from different commercial sources. Liquid amines, except 2-bromopyridine, were distilled twice, first over KOH (10 $\frac{g}{L}$) and second over NaH (10 $\frac{g}{L}$) and stored under argon in headspace vials prior to use. DMAP was ground in a mortar prior to use. The analytes and 2-bromopyridine were purchased from commercial sources and used as received. Literature pK_as and experimentally determined limiting shifts (shifts of the fully protonated as well as the free base species) are given in *table 1* and 2.

DMSO was pre-dried over Na_2SO_4 and then fractionally distilled once under reduced pressure over NaH (25 $\frac{g}{L}$). Acetonitrile was distilled twice, first over NaH (2 $\frac{g}{L}$) and then over P_2O_5 (2 $\frac{g}{L}$). Both solvents were stored under argon in headspace vials.

2. Datapoint selection

For each chemical shift imaging experiment a total of 128 datapoints per analyte and internal standard were collected. However, due to the top and bottom 15-20 slices being at the edge of the NMR detection coil and thus being of inferior quality, those datapoints were neglected. Also, datapoints where both indicators are at their limiting shifts were disregarded as no definitive pH value could be determined from them. Therefore, the number of datapoints varies in each titration.

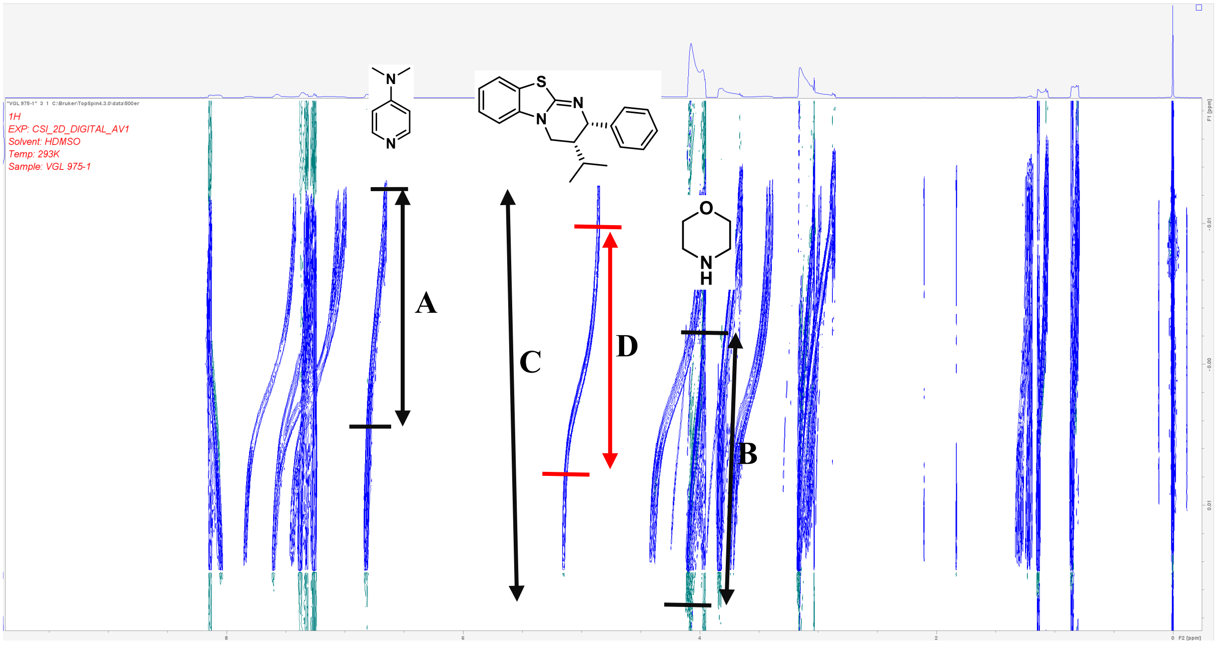

Figure S1: CSI experiment of HyperBTM. Arrow A shows the area for which the pH can be calculated from indicator 1 (DMAP). Arrow B shows the area for indicator 2 (morpholine) from which the pH can be calculated. Arrow C indicates the total area for which the pH can be calculated. Arrow D shows the area which is useful for the pKa determination of HyperBTM. Area D must be contained in area C otherwise only the parts of D which are, can be used for the calculation.

3. Sample preparation

First, a stock solution containing the analyte, two basic indicators and tetramethyl silane was prepared with the respective solvent (DMSO or acetonitrile).

Then 5-10 mg of acid were weighed into a NMR tube and subsequently covered with glass beads.

1)

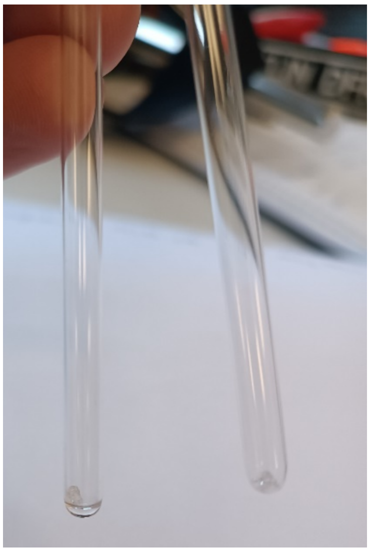

5-10 mg of acid in NMR tube

2)

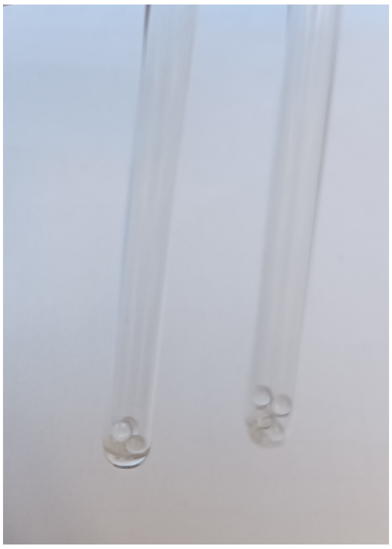

Cover with 1 mm glass beads

Afterwards, 550 µL of the stock solution was layered carefully over the glass beads and the acid. The sample is then put in a 28 °C water bath (for DMSO) for 20 - 24h or left on the bench at 22 °C (for acetonitrile) for 1.5 - 2 h and then the NMR measurement was carried out at 20 °C.

3)

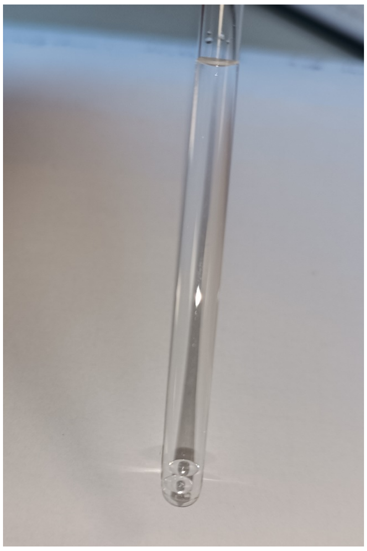

Overlay the acid with the stock solution of two indicators and the analyte

4)

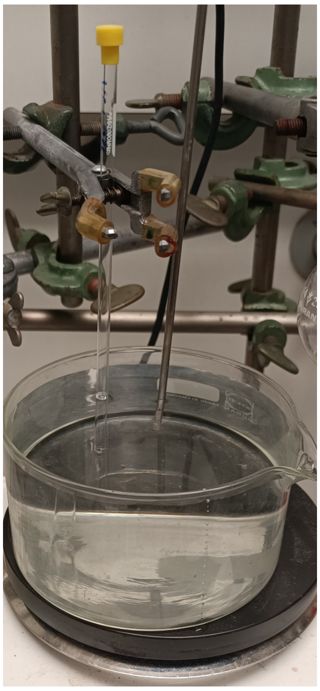

Development of gradients in temperated water bath

5)

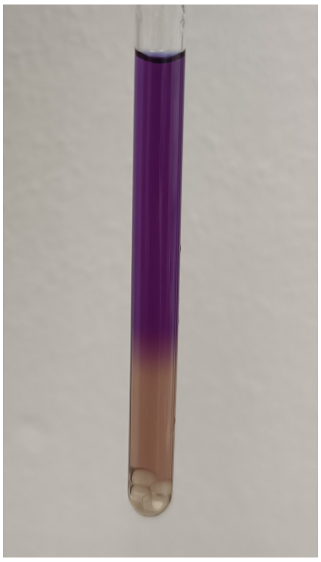

Visualized gradient after 18h in DMSO (with Bromthymolblue)

4. Indicator and acid Data

Table S1: Limiting shifts(δ_H for the fully protonated and δ_L for the free base), pKas and abbreviations of indicators and acids in acetonitrile.

Compound	Abbreviation	Literature pK_a	δ_H / [ppm]	δ_L / [ppm]
Tetramethylsilane	TMS	-	0	0
Morpholine	Morph	16.61 [4]	3.8946	3.5370
4-(dimethylamino)pyridine	DMAP	17.95 [5]	6.81952	6.5503
N-Ethyldiethanolamine	$Et(EtOH)_2N$	18.3	1.2830	0.9993
Pyrrolidine	Pyr	19.56 [6]	3.2364	2.7199
Quinucleidine-3-ol	Quin	18.7 [7]	4.1270	3.8146
2,4-Dinitrobenzoic acid	DNB	16.6 [8]	-	-
Chloroacetic acid	ClAc	15.34 [9]	-	-

Table S2: Limiting shifts(δ_H for the fully protonated and δ_L for the free base), pKas and abbreviations of indicators and acids in DMSO.

Compound	Abbreviation	Literature pK_a	δ_H / [ppm]	δ_L / [ppm]
Triethylamine	Et_3N	9 [10]	1.1886	0.9257
Tetramethylsilane	TMS	-	0	0
Morpholine	Morph	9.2 [11]	3.7728	3.4879
2-Bromopyridine	2-brompyr	7.02 [12]	8.5533	8.2461
N,N-Dimethylbenzylamine	DMBA	7.6 [10]	4.2760	3.4026
4-(dimethylamino)pyridine	DMAP	7.91 [12]	6.98095	6.578
Meldrums acid	Mel	7.3 [13]	-	-
2,4-Dinitrobenzoic acid	DNB	6.5 [14]	-	-

5. Titration Data

5.1. Acetonitrile

Table S3: Determined pK_as of analytes and titration data in acetonitrile

Catalyst	Determined pKa	Mass of acid / [g]	δ_H / [ppm]	δ_L / [ppm]	Titration components / [mM]*
DHBP	17.5	0.0087 ClAc	7.8165	7.3530	10 DHBP, 20 DMAP, 20 $Et(EtOH)_2N$, 20 TMS
HyperBTM	16.9	0.0068 ClAc	5.0082	4.8337	10 HyperBTM, 10 DMAP, 10 Morph, 20 TMS
Tetramisole	17.2	0.0075 ClAc	5.6577	5.3279	10 TM, 20 DMAP, 20 $Et(EtOH)_2N$, 20 TMS
HBTM	17.9	0.0077 ClAc	4.9924	4.6104	10 HBTM, 10 DMAP, 10 $Et(EtOH)_2N$, 20 TMS
BTM	16.8	0.0070 ClAc	4.9198	4.3250	10 BTM, 10 DMAP, 10 Morph, 20 TMS
Quinine	18.8	0.0078 DNB	6.1680	5.2754	10 Quinine, 20 $Et(EtOH)_2N$, 20 Pyr, 20 TMS
Quinidine	19.0	0.0075 DNB	6.2530	5.3438	10 Quinidine, 20 $Et(EtOH)_2N$, 20 Pyr, 20 TMS
Cinchonine	18.4	0.0070 DNB	6.3300	5.3704	10 Cinchonine, 20 $Et(EtOH)_2N$, 20 Pyr, 20 TMS
Cinchonidine	18.4	0.0081 DNB	6.2480	5.3338	10 Cinchonidine, 20 $Et(EtOH)_2N$, 20 Pyr, 20 TMS
$Et(EtOH)_2N$	18.3	0.0074 ClAc	1.2830	0.9993	20 $Et(EtOH)_2N$, 20 DMAP, 20 Quin

*Based on the accuracy of weighing in, small deviations are possible.

5.2. DMSO

Table S4:Determined pK_as of analytes and titration data in DMSO.

Catalyst	Determined pK_a	Mass of acid / [g]	δ_H / [ppm]	δ_L / [ppm]	Titration components / [mM]*
DHBP	7.8	0.0075 MeI	4.1886	3.7776	10 DHBP, 10 DMAP, 10 Et_3N, 20 TMS
HyperBTM	7.4	0.0088 MeI	5.1961	4.8047	10 HyperBTM, 20 DMAP, 20 Morph, 20 TMS
Tetramisole	7.2	0.0069 DNB	5.7359	5.3196	10 TM, 20 DMAP, 20 Et_3N, 20 TMS
HBTM	7.3	0.0072 MeI	5.0156	4.6324	10 HBTM, 10 DMAP, 10 Et_3N, 20 TMS
BTM	6.3	0.0070 DNB	4.8800	4.3720	10 BTM, 20 2-Brompyr, 20 DMBA, 20 TMS
Quinine	8.4	0.0080 MeI	5.9426	5.2374	10 Quinine, 20 DMAP, 20 Et_3N, 20 TMS
Quinidine	8.5	0.0078 MeI	6.0257	5.2726	10 Quinidine, 20 DMAP, 20 Et_3N, 20 TMS
Cinchonine	8.2	0.0074 MeI	6.0468	5.2935	10 Cinchonine, 20 DMAP, 20 Et_3N, 20 TMS
Cinchonidine	8.2	0.0079 MeI	5.9418	5.2597	10 Cinchonidine, 20 DMAP, 20 Et_3N, 20 TMS

*Based on the accuracy of weighing in, small deviations are possible.

6. References

[1] Wallace M.; Adams D. J.; Iggo J. A., *Anal. Chem.* **(2018)**, 90, 4160-4166.
[2] Schenck G., Baj K., Iggo J. A., Wallace M., *Anal. Chem.* **(2022)**, 94, 8115-8119
[3] Hwang T. L., Shaka A. J., *J. Magn. Reson., Series A* **(1995)**, 112, 275-279
[4] Coetzee J. F., Padmanabhan G. R., *J. Am. Chem. Soc.* **(1965)**, 87, 22, 5005–5010
[5] Kaljurand I., Kütt A., Sooväli L., Rodima T., Mäemets V., Leito I., Koppel I. A., *J. Org. Chem.* **(2005)**, 70, 3, 1019–1028
[6] Searles S., Tamres M., Block F., Quarterman L. A., *J. Am. Chem. Soc.* **(1956)**, 78, 19, 4917–4920
[7] Beltrame P., Gelli G., Loi A., *Gazetta Chimica Acta* **(1980)**, 110, 491-494
[8] Leesment A., Selberg S., Tammiste M., Vu A. H., Nguyen T. H., Taylor-King L., Leito I., *Anal. Chem.* **(2022)**, 94, 9, 4059–4064
[9] Kolthoff I. M., Chantooni M. K., *J. Chem. Eng. Data* **(1999)**, 44, 1, 124–129
[10] Kolthoff I. M., Chantooni Jr. M. K., Bhowmik S., *J. Am. Chem. Soc.* **(1968)**, 90, 1, 23–28
[11] Crampton R. M., Robotham I. A., *J. Chem. Res. (S)* **(1997)**, 22-23
[12] Carabias-Martínez R., Rodríguez-Gonzalo E., Domínguez-Alvarez J., Miranda-Cruz E., *Anal. Chim. Acta* **(2007)**, 584, 410-418
[13] Arnett E. M., Maroldo S. G., Schilling S. L., Harrelson J. A., *J. Am. Chem. Soc.* **(1984)**, 106, 22, 6759–6767
[14] Maran F., Celadon D., Severin M. G., Vianello E., *J. Am. Chem. Soc.* **(1991)**, 113, 24, 9320–9329

7. NMR Spectra:

7.1. Acetonitrile

7.1.1. Quinidine:

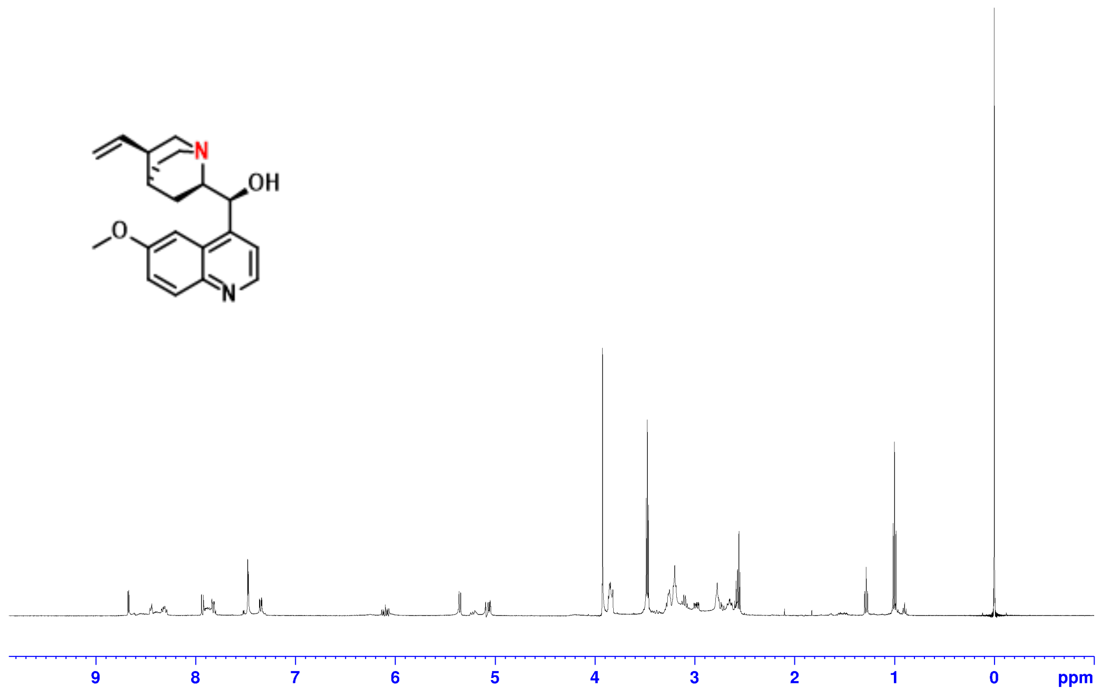

Figure S2: ¹H of CSI experiment over whole sample for Quinidine in acetonitrile.

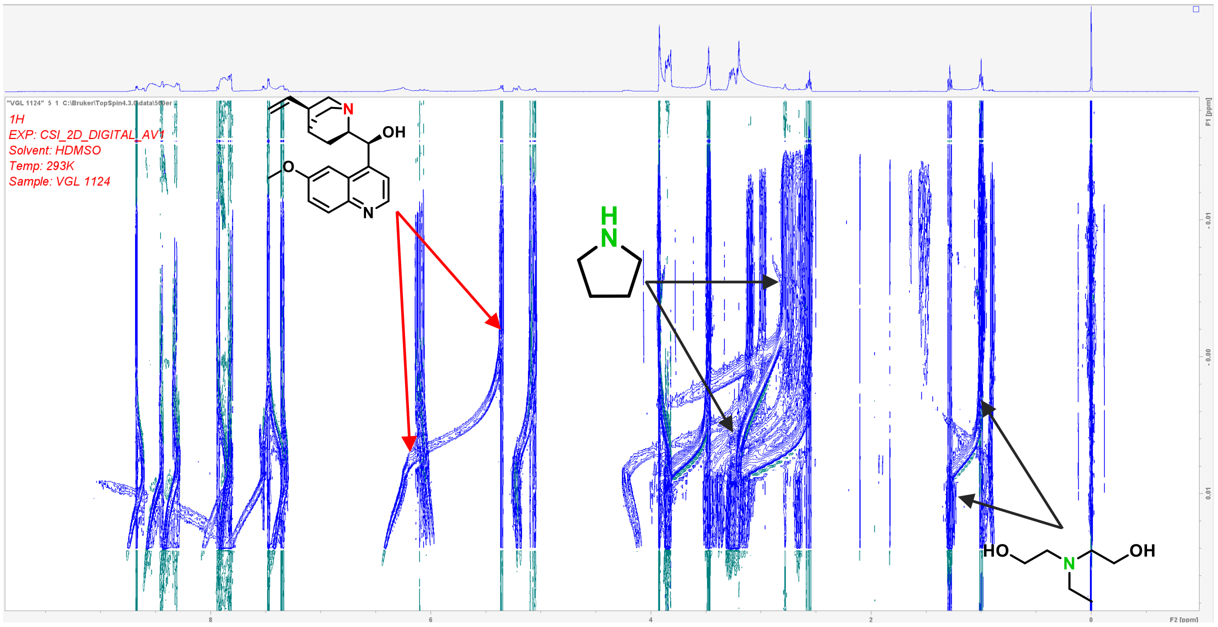

Figure S3: CSI experiment for Quinidine in acetonitrile.

7.1.2. Cinchonine:

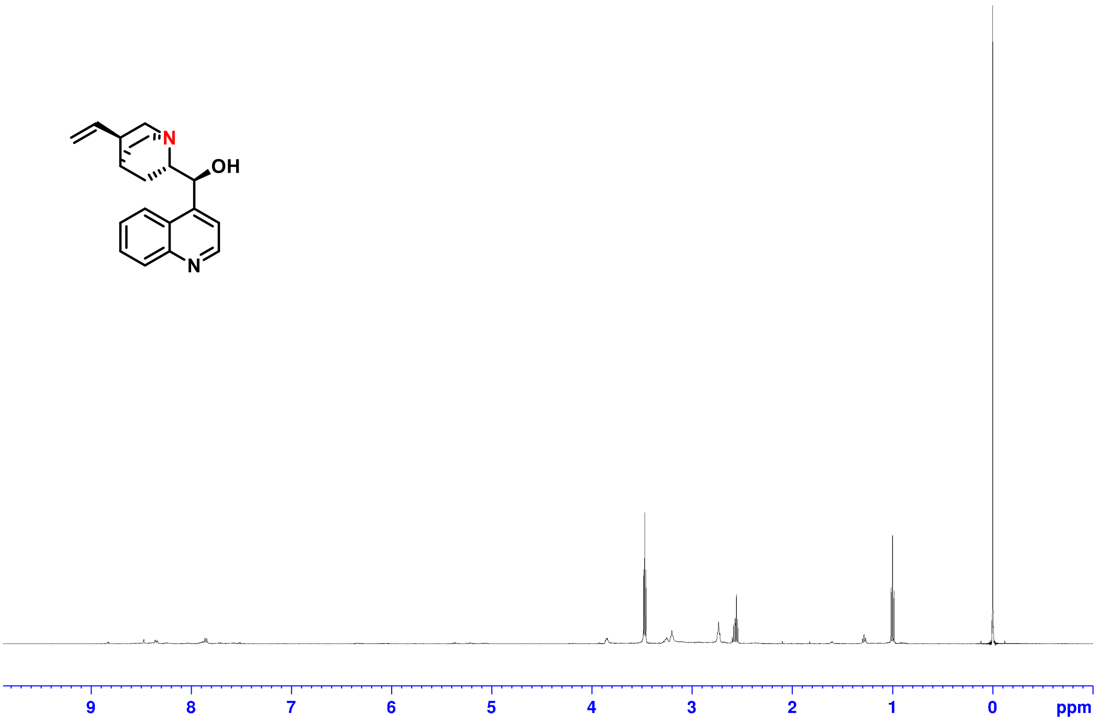

Figure S4: [1]H experiment over whole sample of CSI experiment of Cinchonine in acetonitrile.

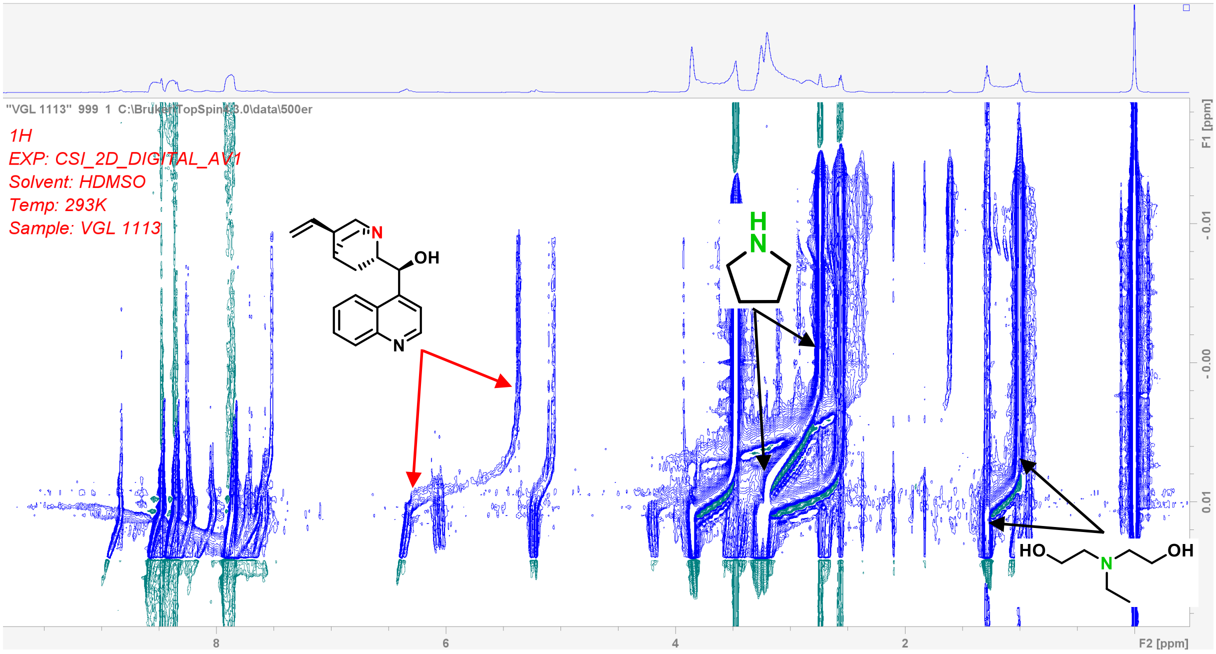

Figure S5: CSI experiment for Cinchonine in acetonitrile.

7.1.3. Cinchonidine:

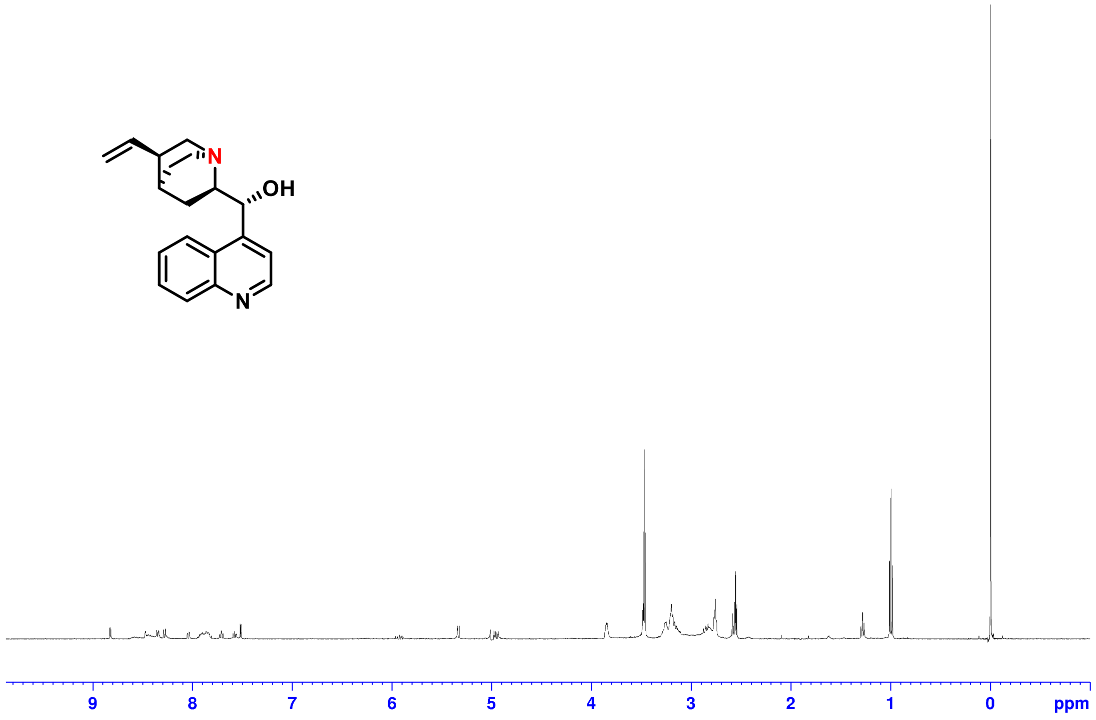

Figure S6: [1]H experiment over whole sample of CSI experiment of Cinchonidine in acetonitrile.

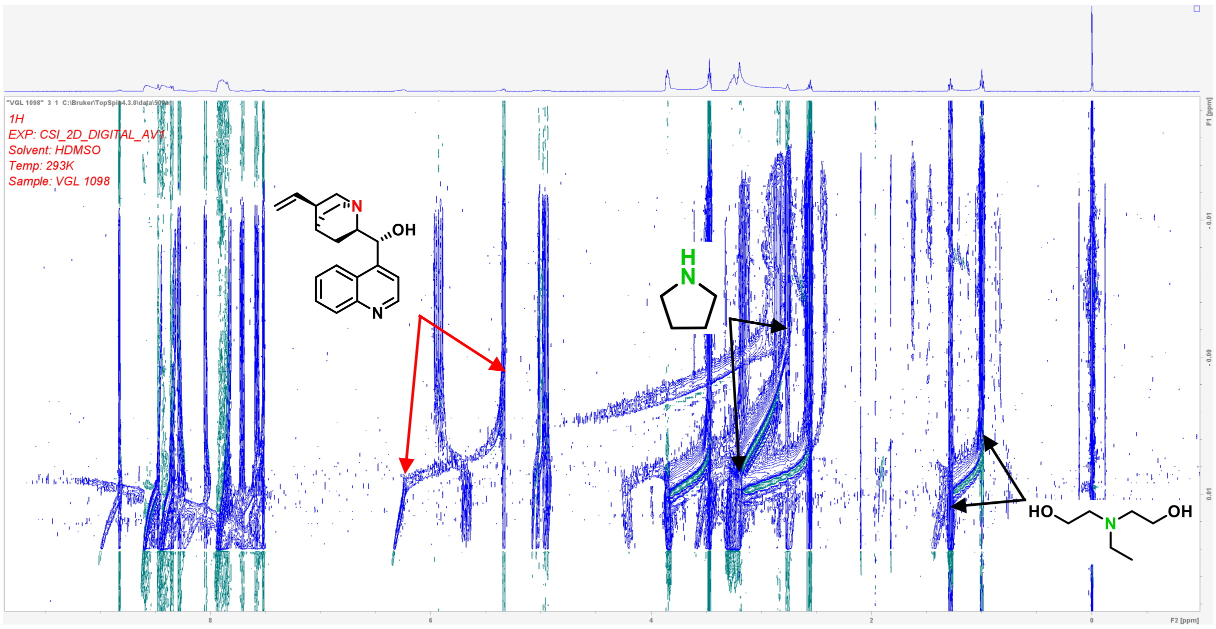

Figure S7: CSI experiment for Cinchonidine in acetonitrile.

7.1.4. Quinine:

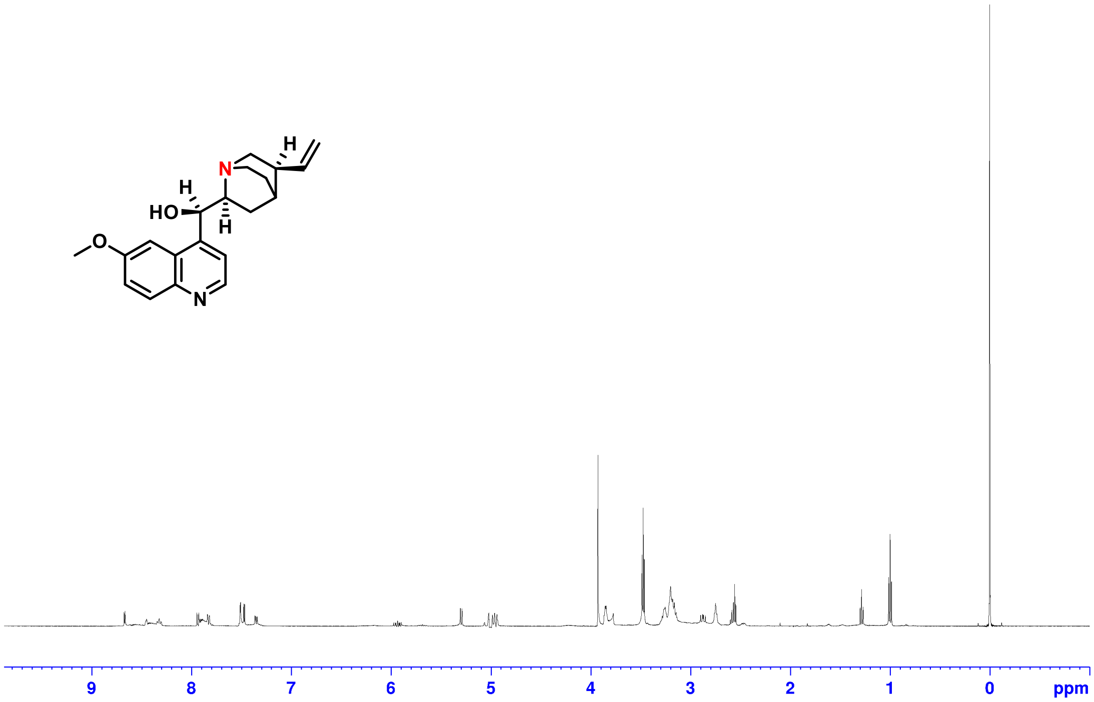

Figure S8: [1]H experiment over whole sample of CSI experiment of Quinine in acetonitrile.

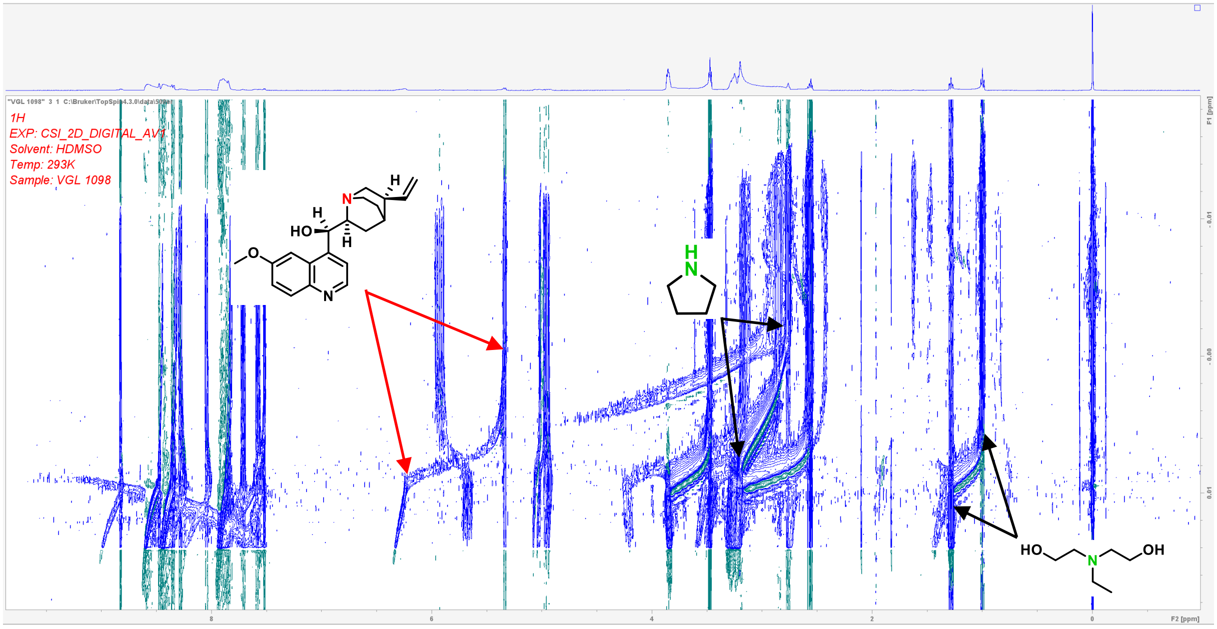

Figure S9: CSI experiment for Quinine in acetonitrile.

7.1.5. N-Ethyldiethanolamine:

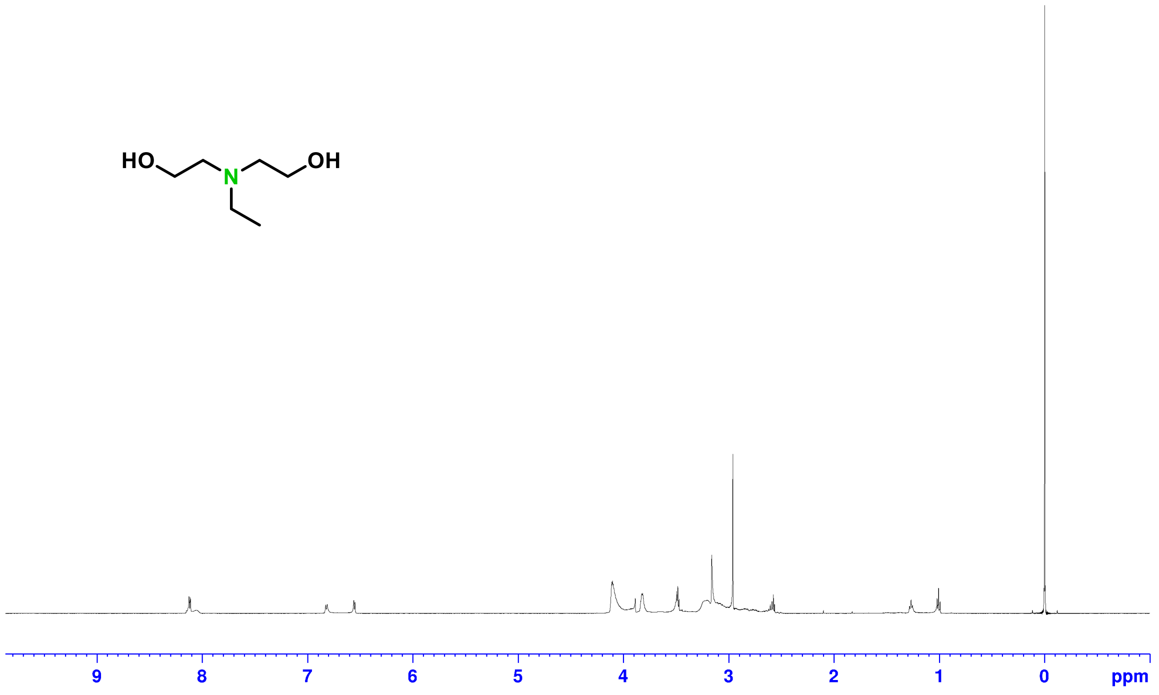

Figure S10: ¹H experiment over whole sample of CSI experiment of N-ethyldiethanolamine in acetonitrile.

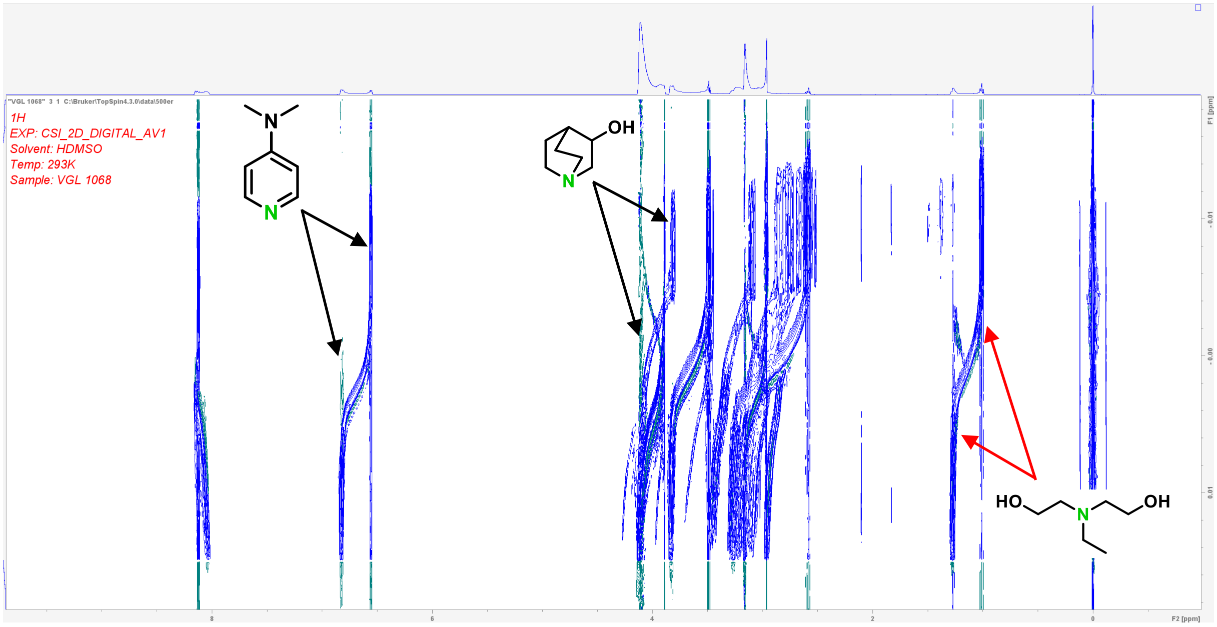

Figure S11: CSI experiment for N-ethyldiethanolamine in acetonitrile.

7.1.6. Tetramisole:

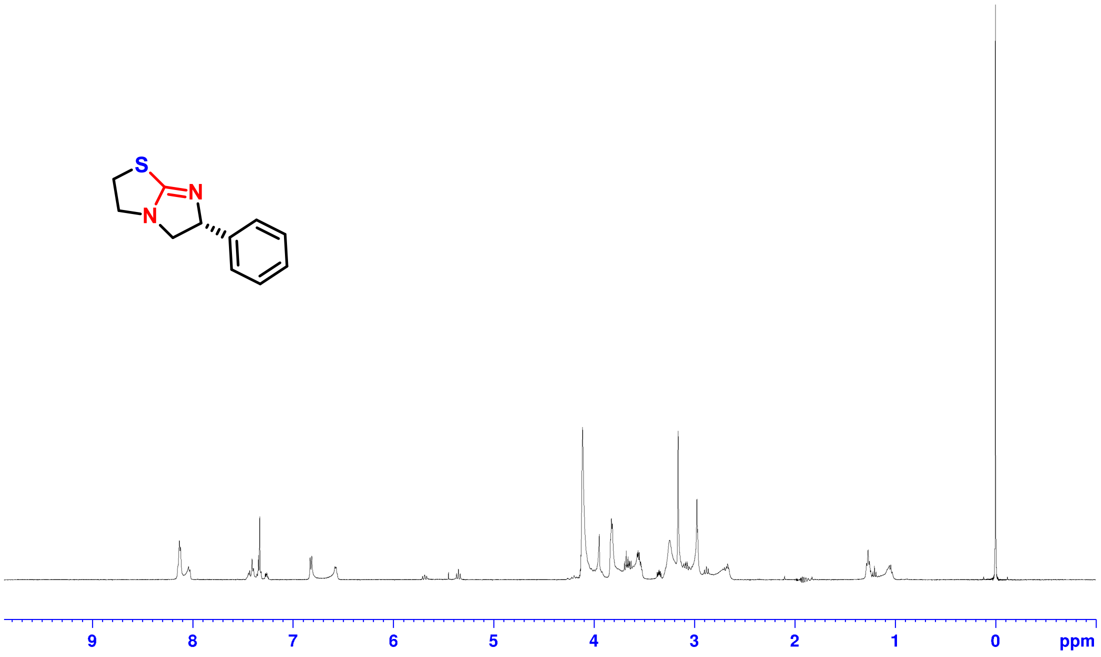

Figure S12: [1]H experiment over whole sample of CSI experiment of Tetramisole in acetonitrile.

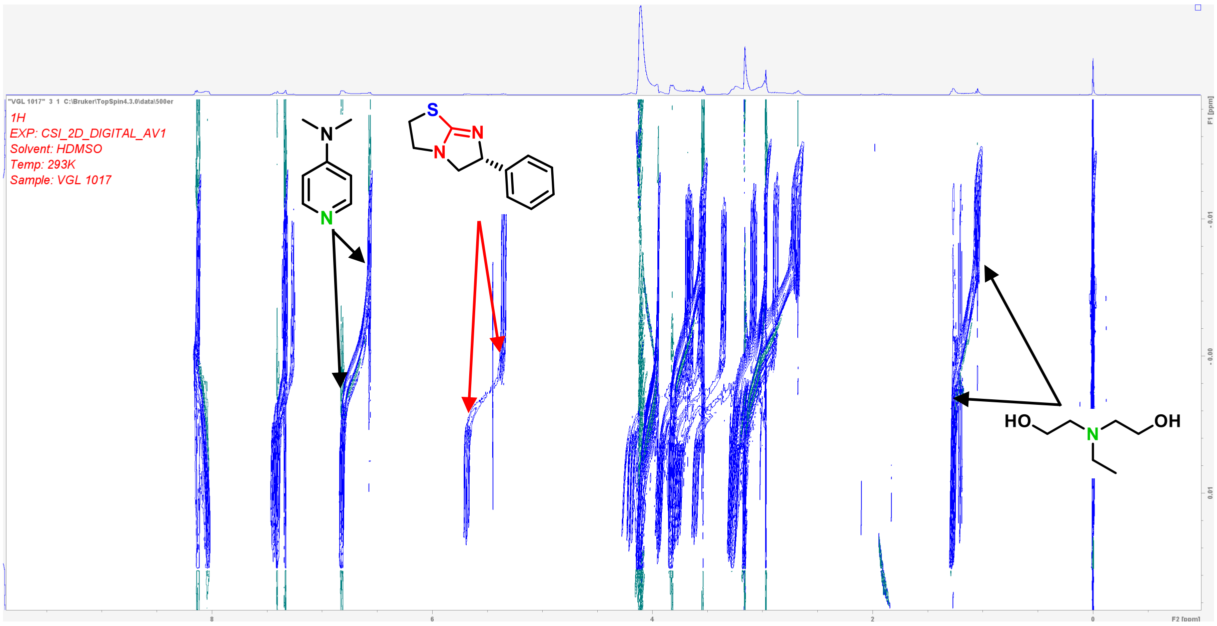

Figure S13: CSI experiment for Tetramisole in acetonitrile.

7.1.7. HBTM:

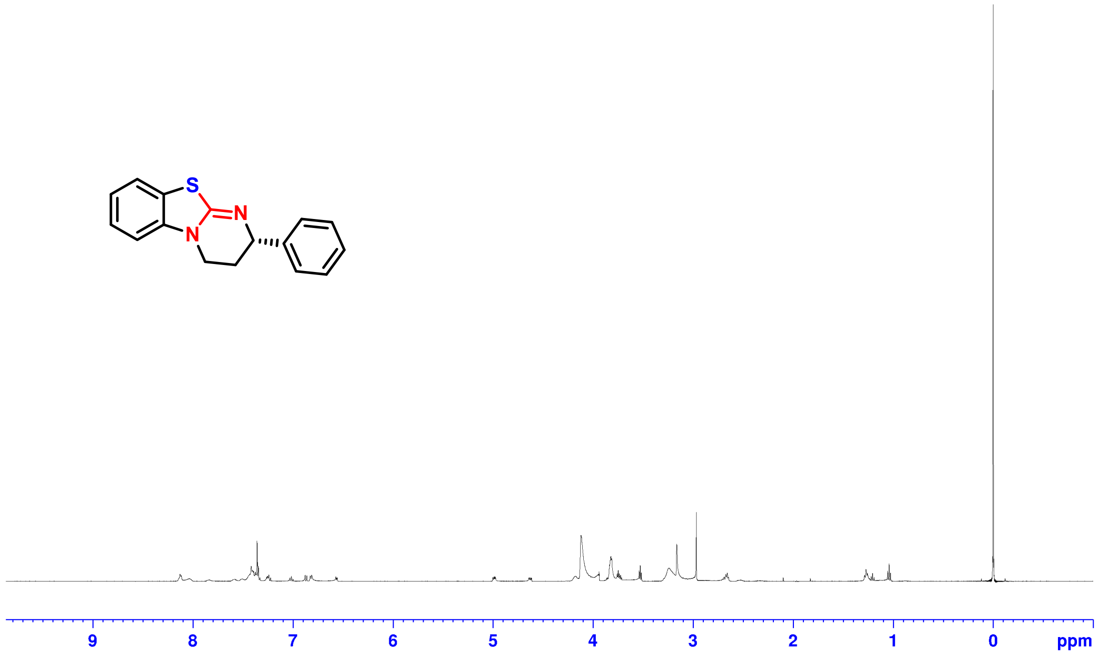

Figure S14: [1]H experiment over whole sample of CSI experiment of HBTM in acetonitrile.

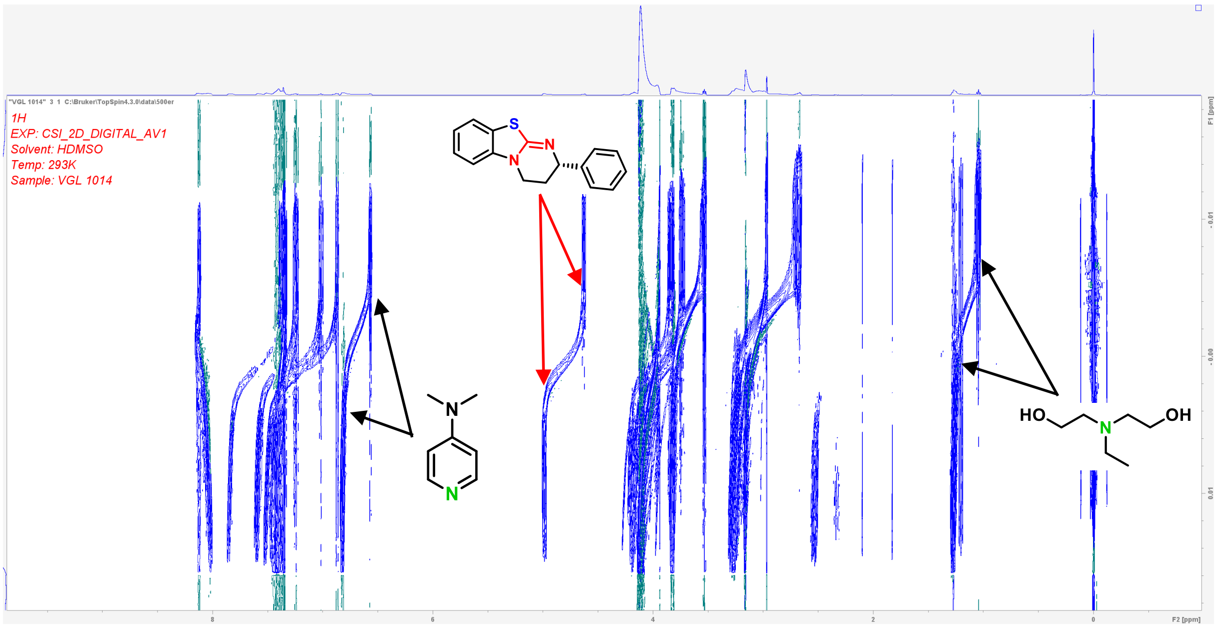

Figure S15: CSI experiment for HBTM in acetonitrile.

7.1.8. DHBP:

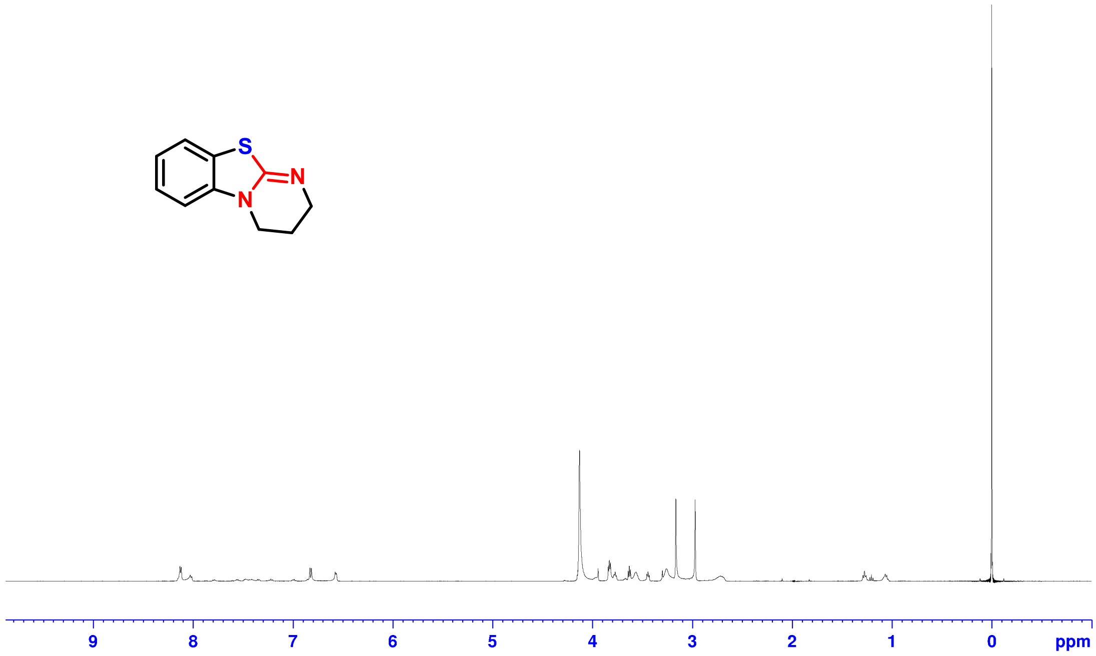

Figure S16: [1]H experiment over whole sample of CSI experiment of DHBP in acetonitrile.

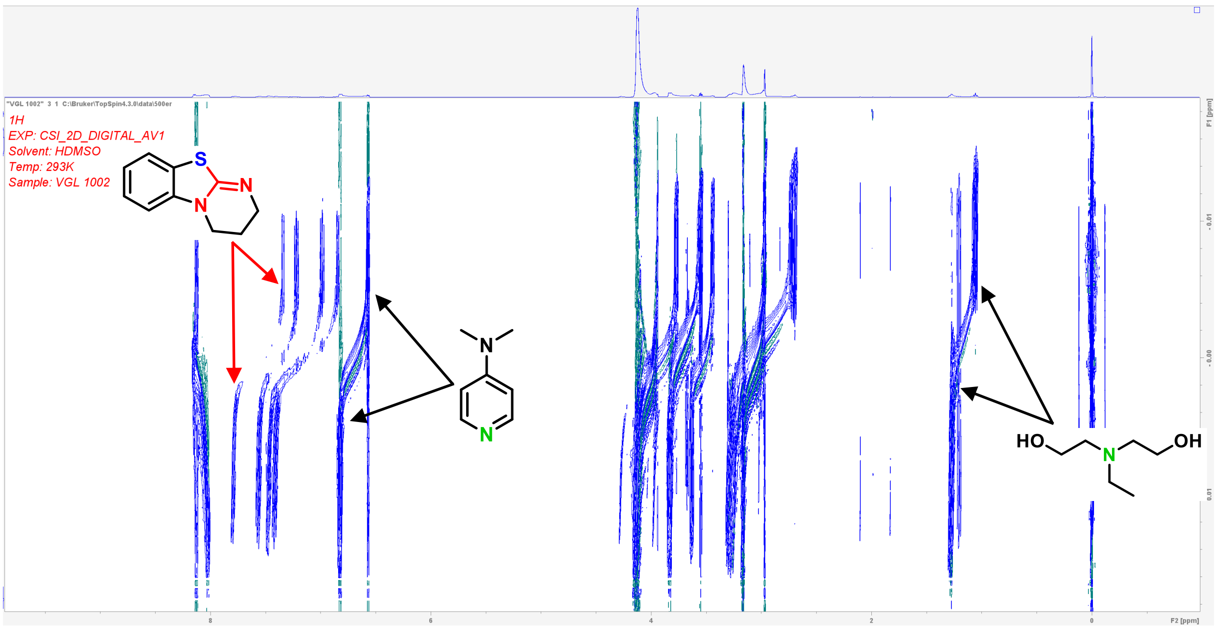

Figure S17: CSI experiment for DHBP in acetonitrile.

7.1.9. BTM:

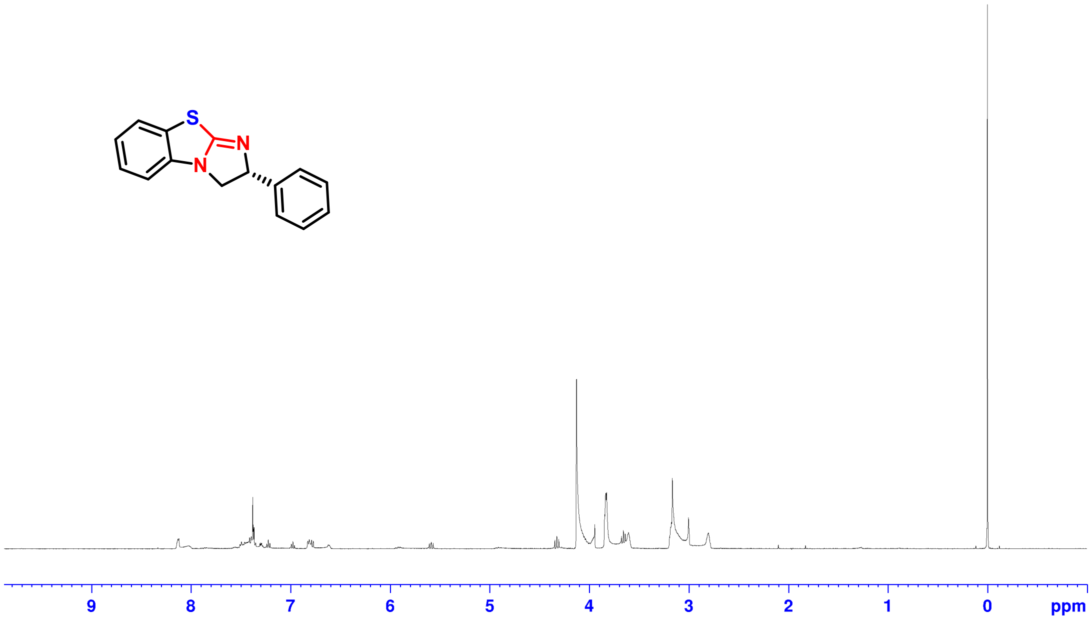

Figure S18: [1]H experiment over whole sample of CSI experiment of BTM in acetonitrile.

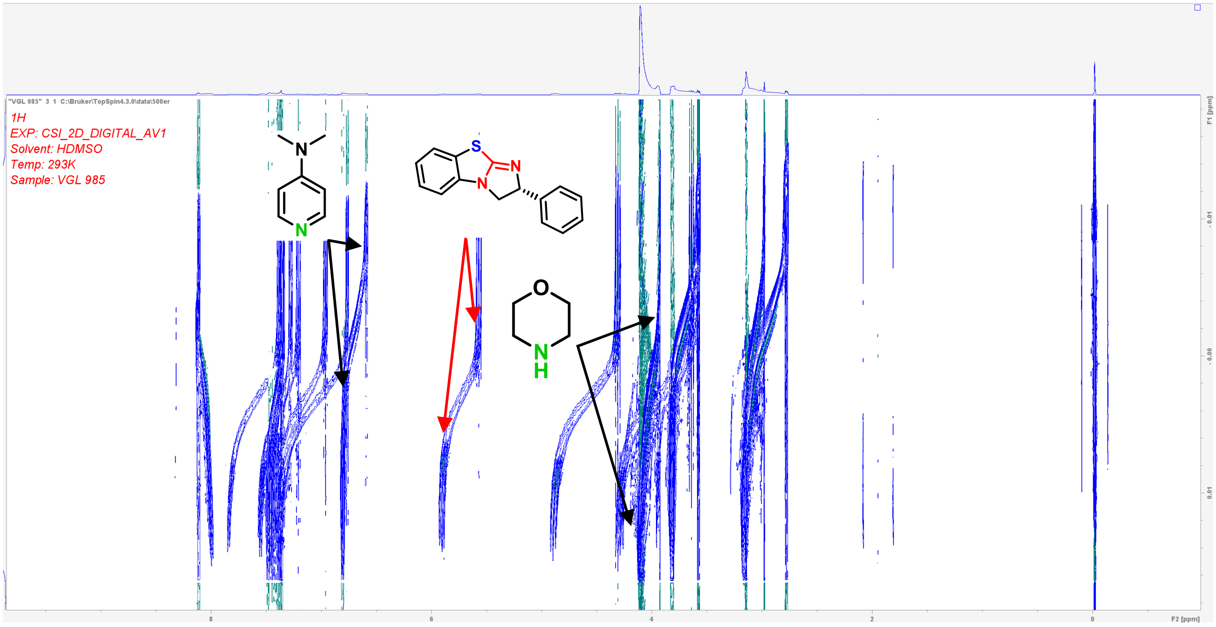

Figure S19: CSI experiment for BTM in acetonitrile.

7.1.10. HyperBTM:

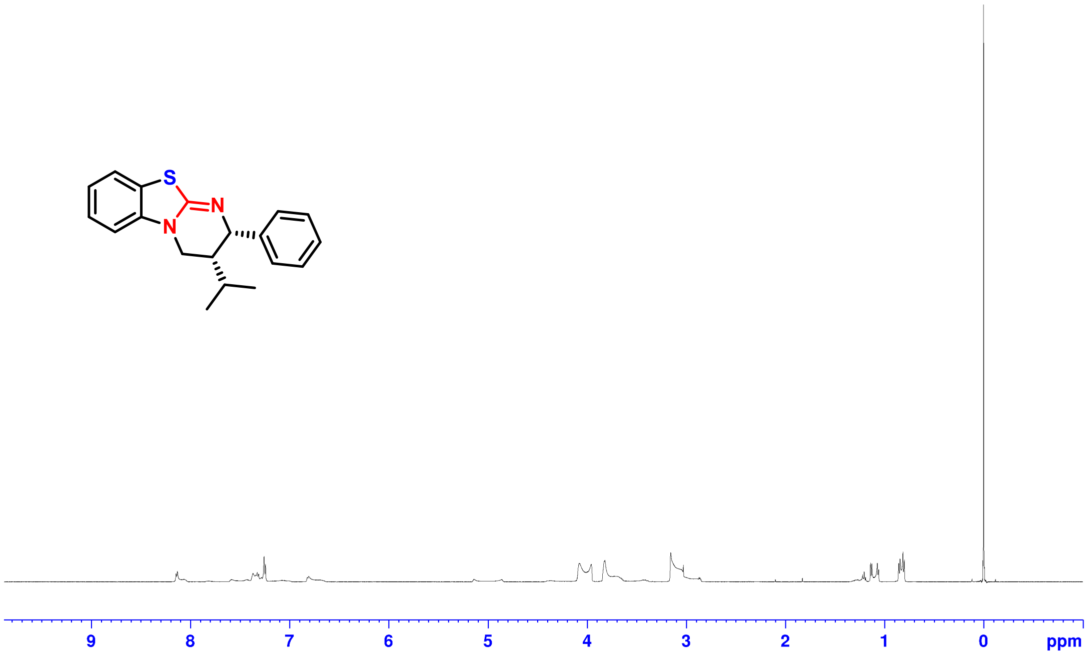

Figure S20: [1]H experiment over whole sample of CSI experiment of HyperBTM in acetonitrile.

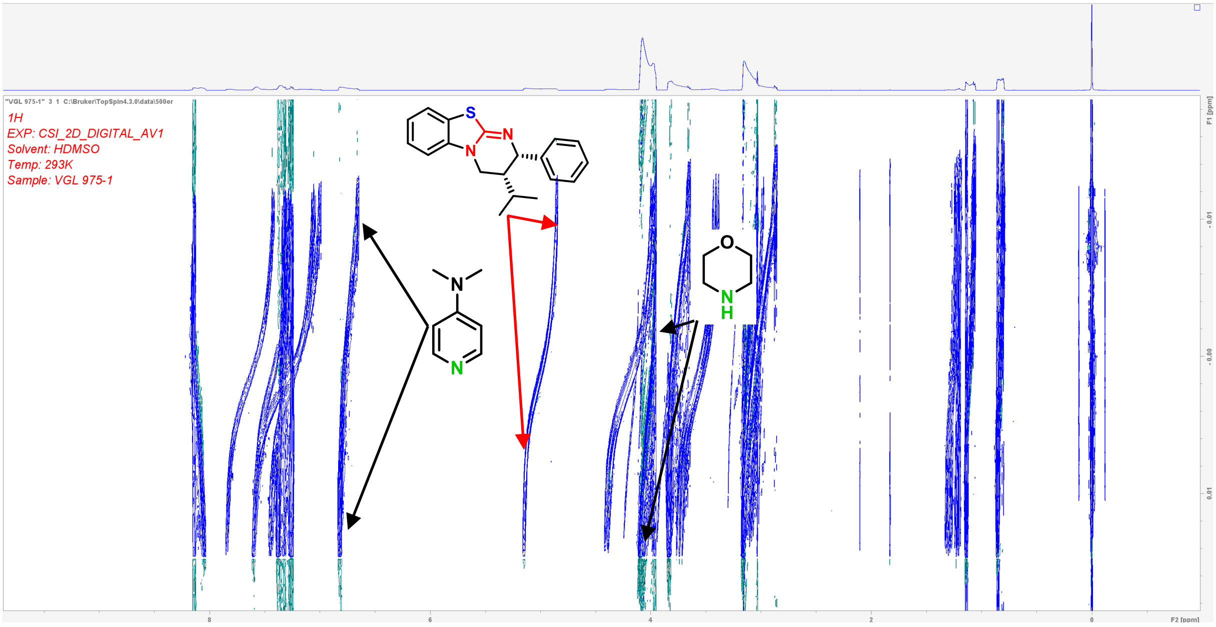

Figure S21: CSI experiment for HyperBTM in acetonitrile.

7.2. DMSO:

7.2.1. BTM:

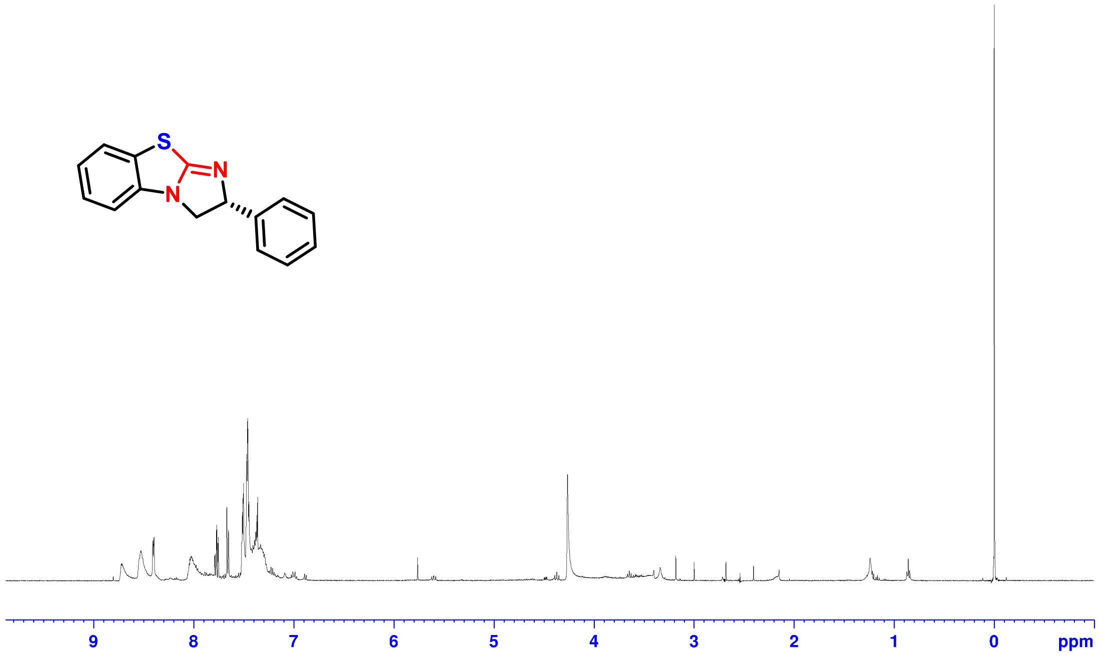

Figure S22: ^{1}H experiment over whole sample of CSI experiment of BTM in DMSO.

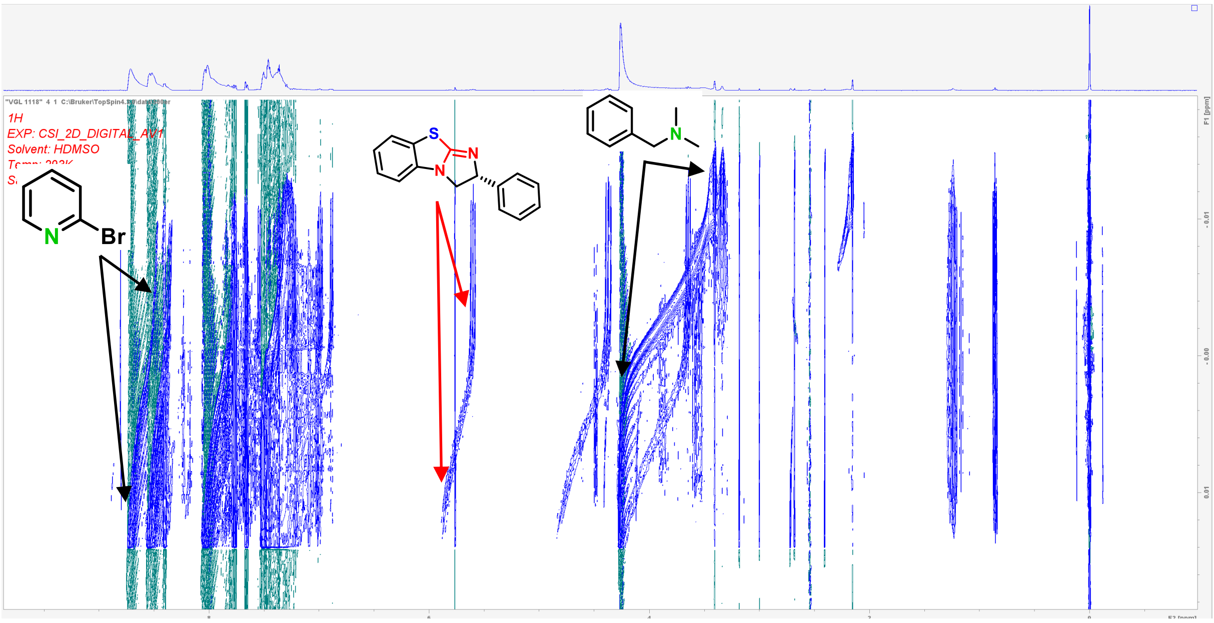

Figure S23: CSI experiment for BTM in DMSO.

7.2.2. Cinchonine:

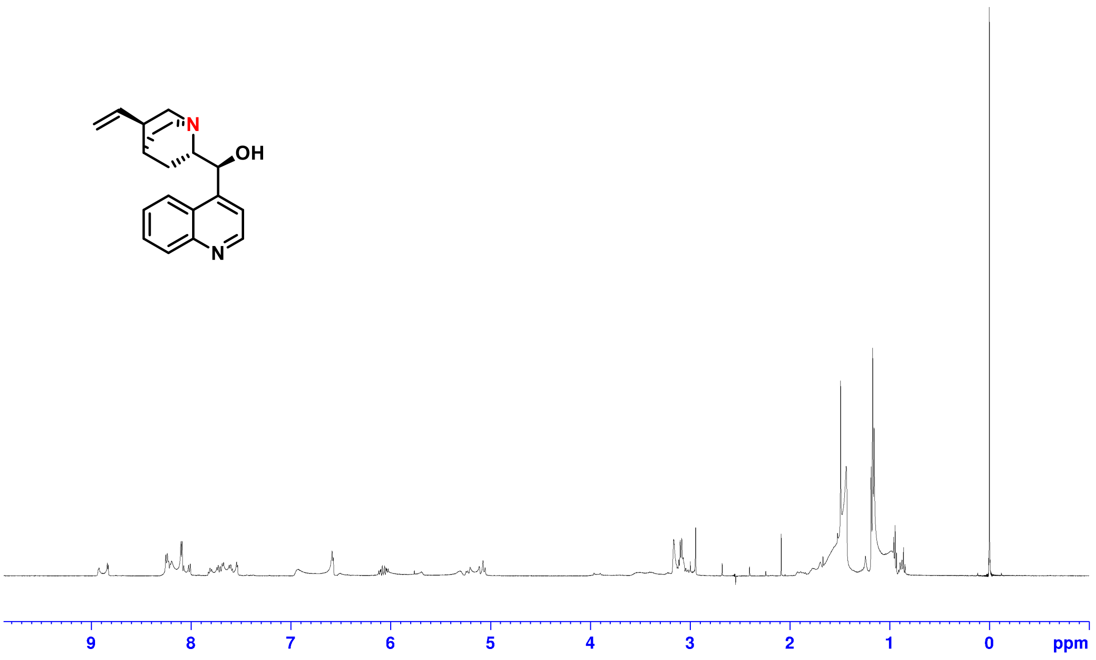

Figure S24: ¹H experiment over whole sample of CSI experiment of Cinchonine in DMSO.

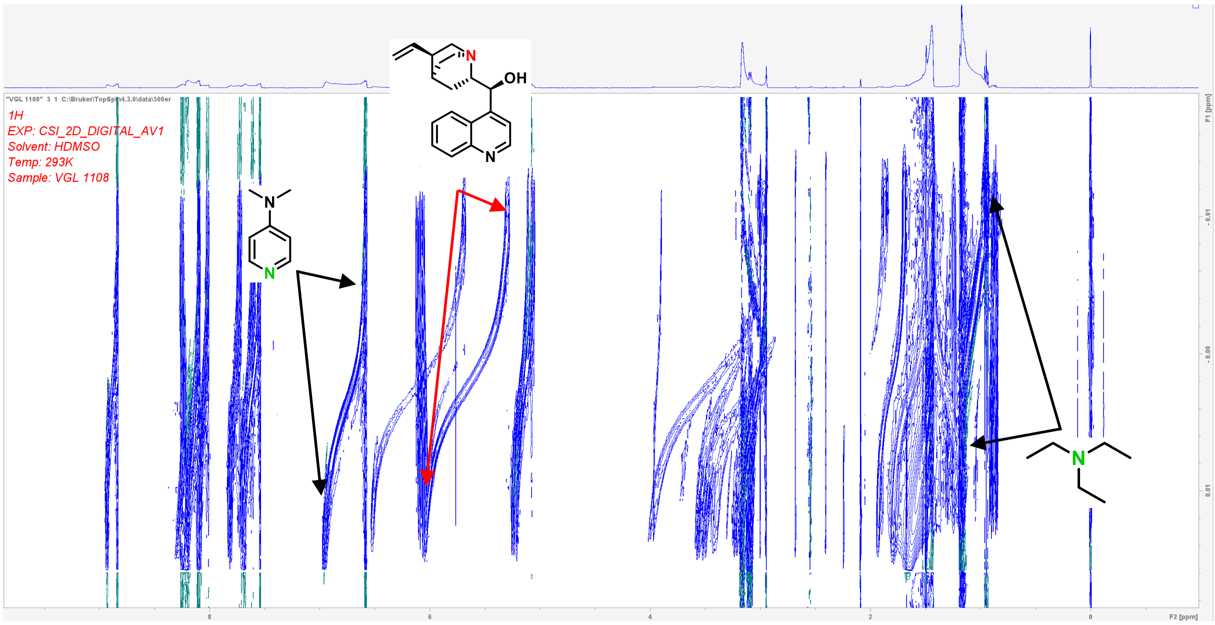

Figure S25: CSI experiment for Cinchonine in DMSO.

7.2.3. Cinchonidine:

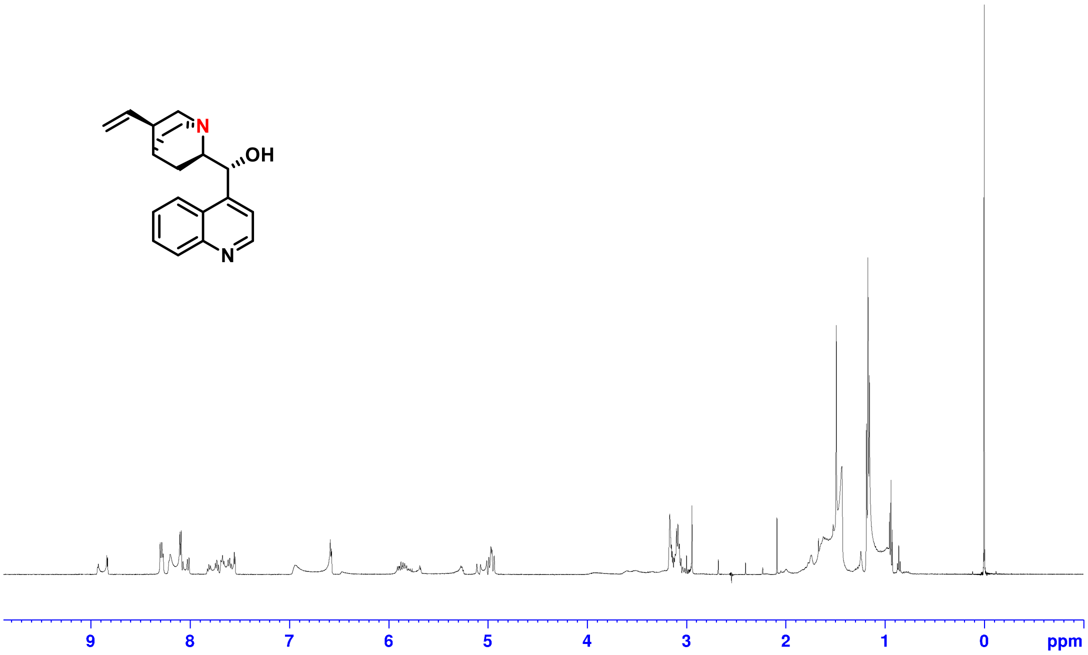

Figure S26: ^{1}H experiment over whole sample of CSI experiment of Cinchonidine in DMSO.

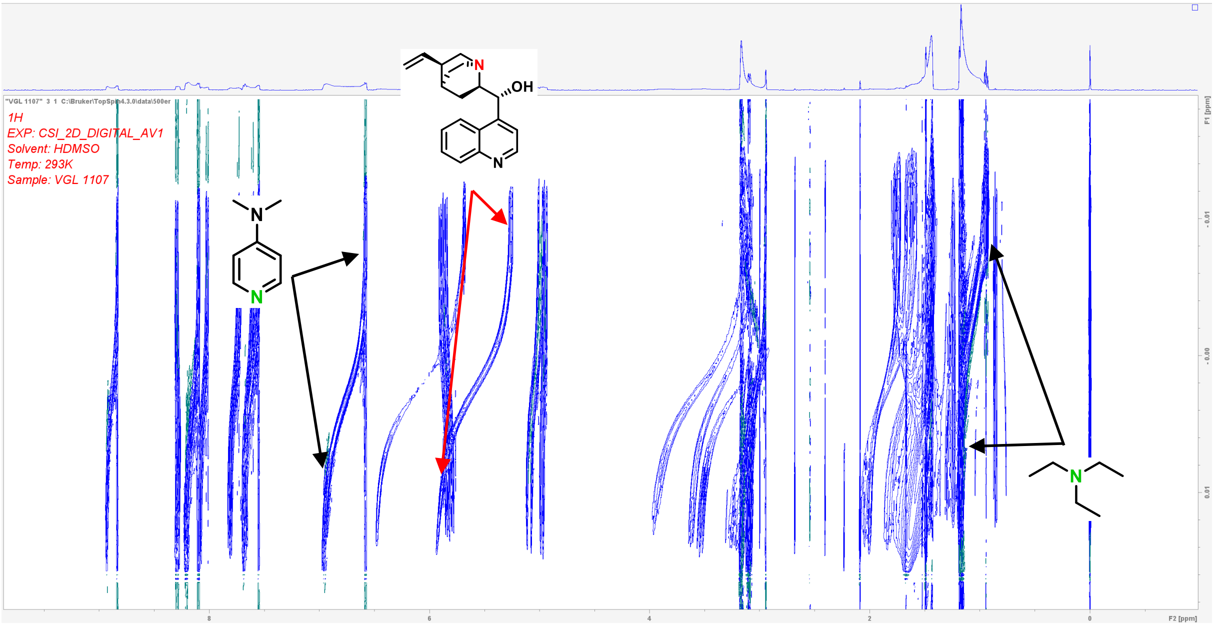

Figure S27: CSI experiment for Cinchonidine in DMSO.

7.2.4. Quinidine:

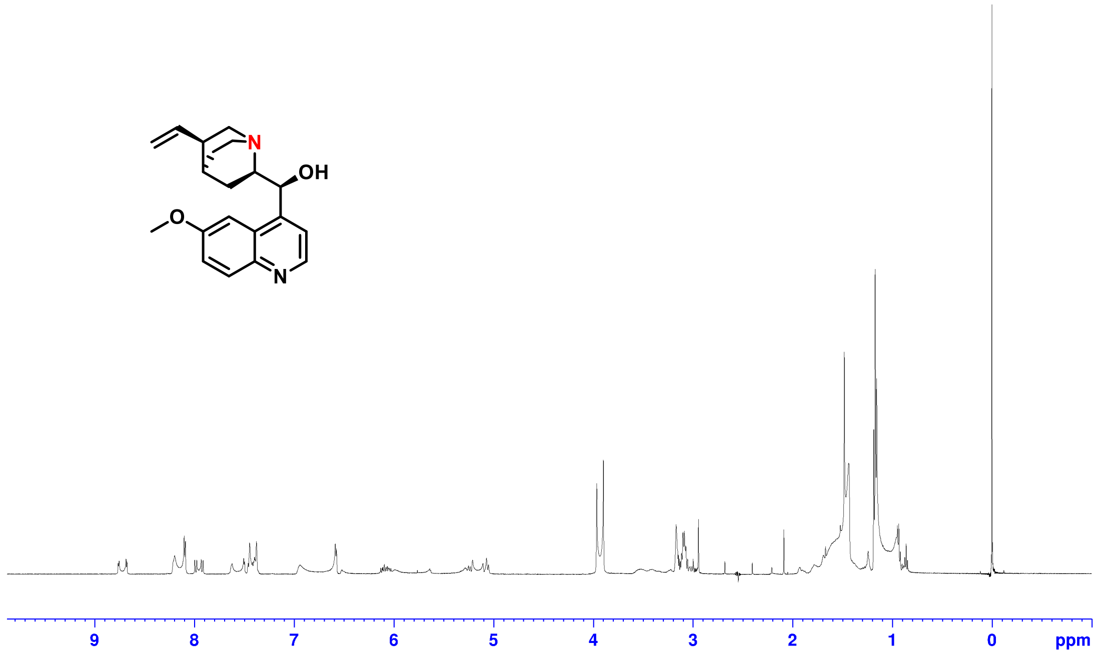

Figure S28: ¹H experiment over whole sample of CSI experiment of Quinidine in DMSO.

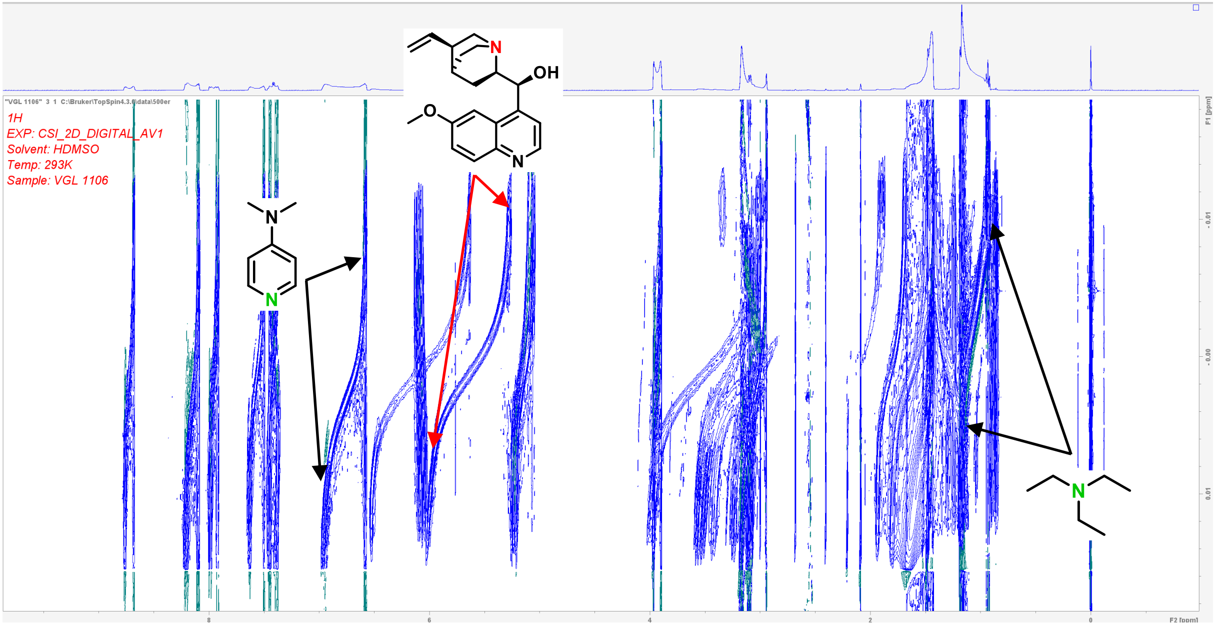

Figure S29: CSI experiment for Quinidine in DMSO.

7.2.5. Quinine:

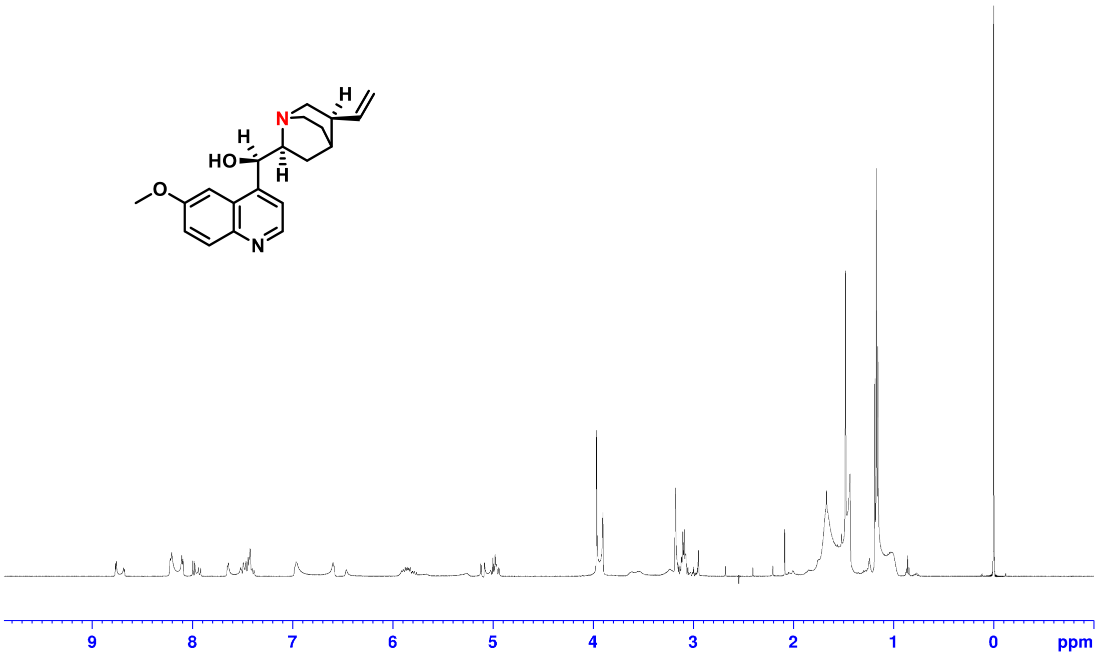

Figure S30: [1]H experiment over whole sample of CSI experiment of Quinine in DMSO.

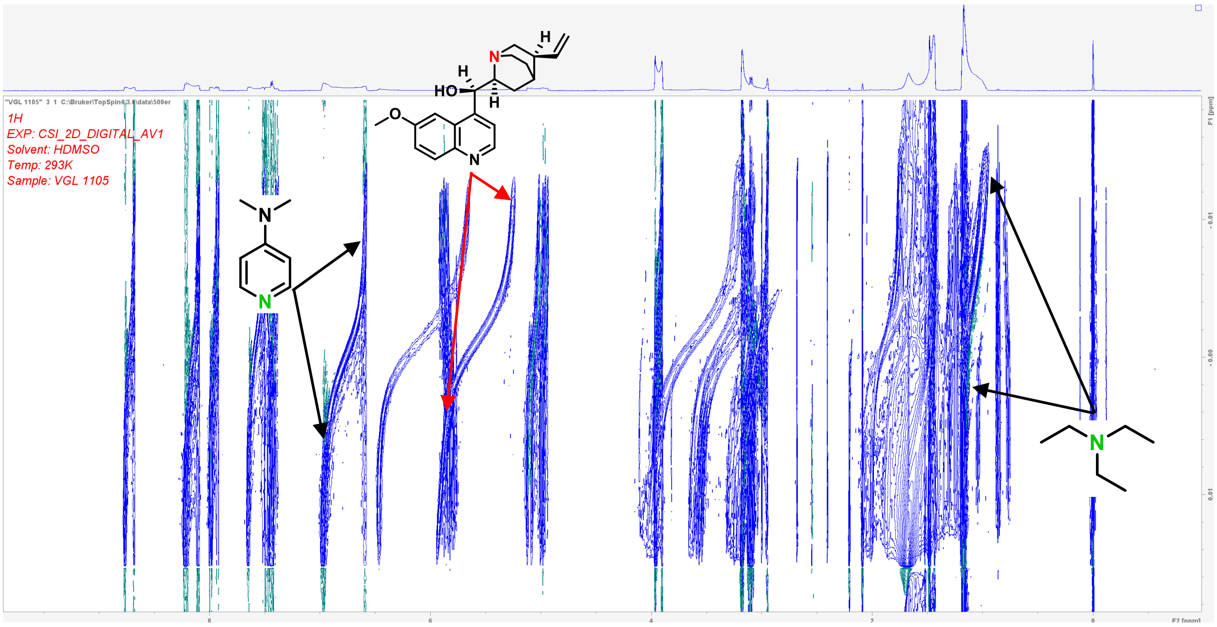

Figure S31: CSI experiment for Quinine in DMSO.

7.2.6. Tetramisole:

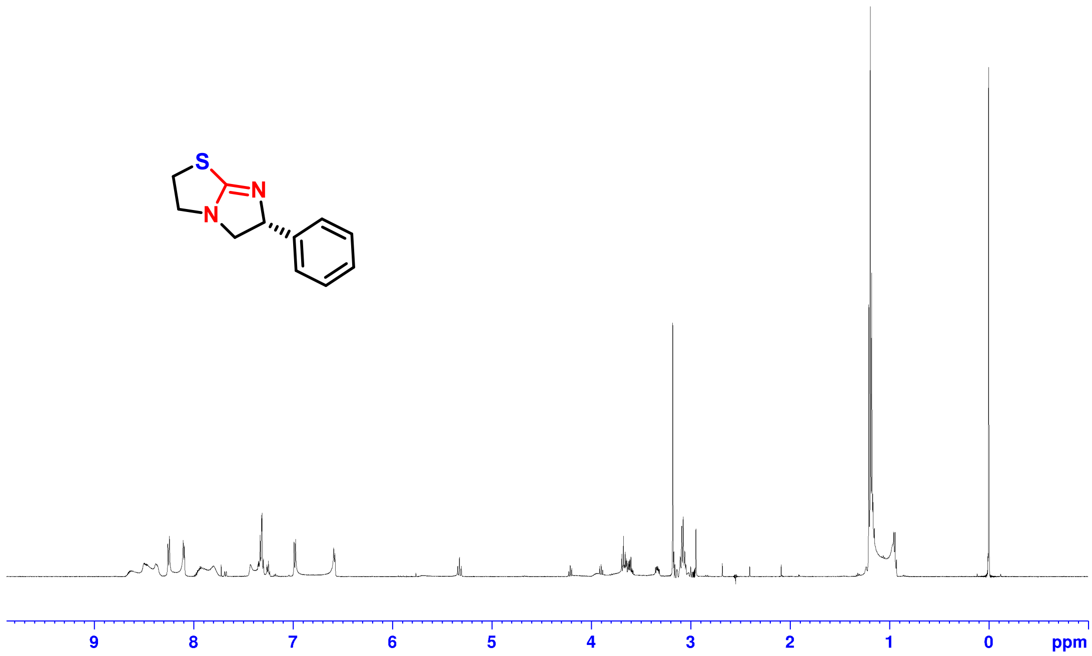

Figure S32: [1]H experiment over whole sample of CSI experiment of Tetramisole in DMSO.

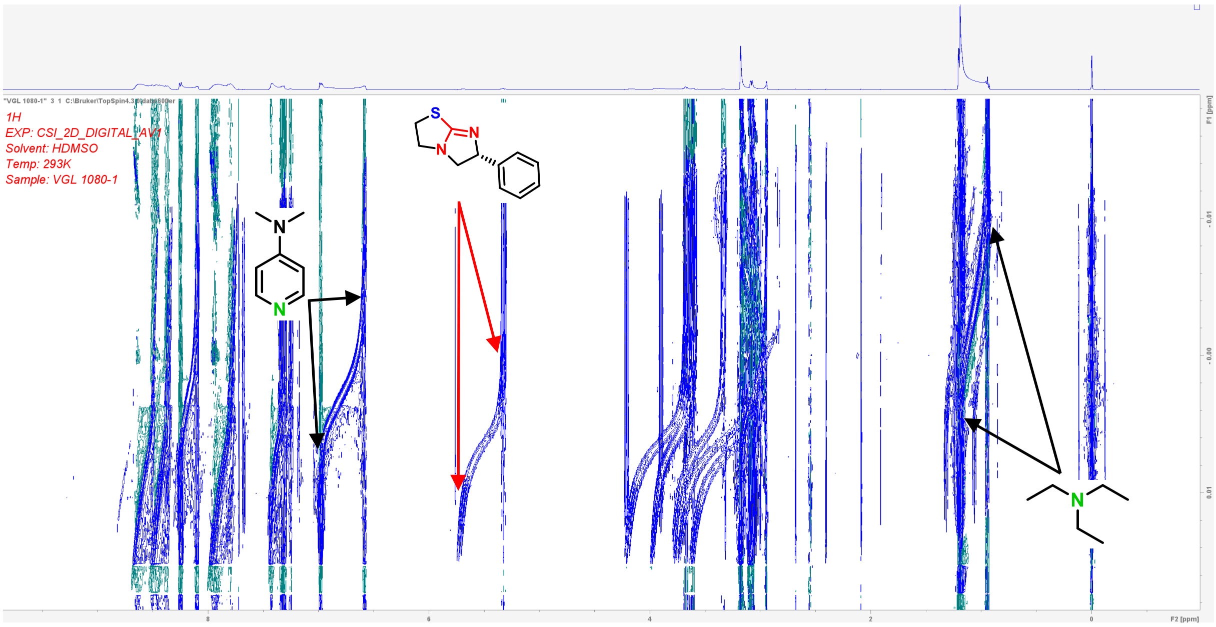

Figure S33: CSI experiment for Tetramisole in DMSO.

7.2.7. HBTM:

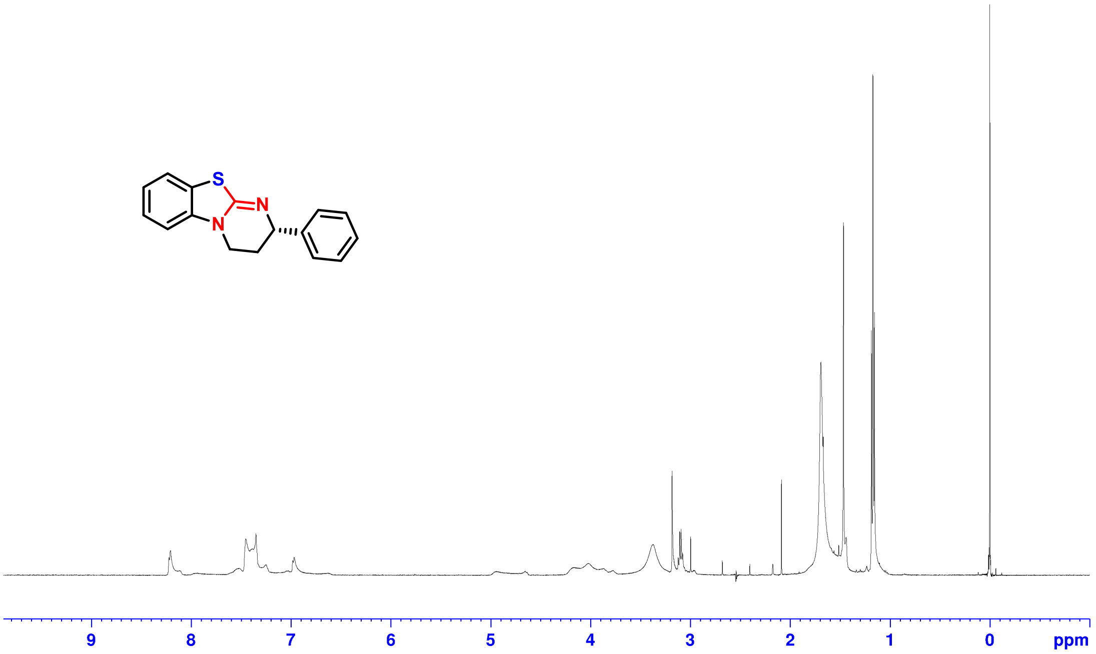

Figure S34: [1]H experiment over whole sample of CSI experiment of HBTM in DMSO.

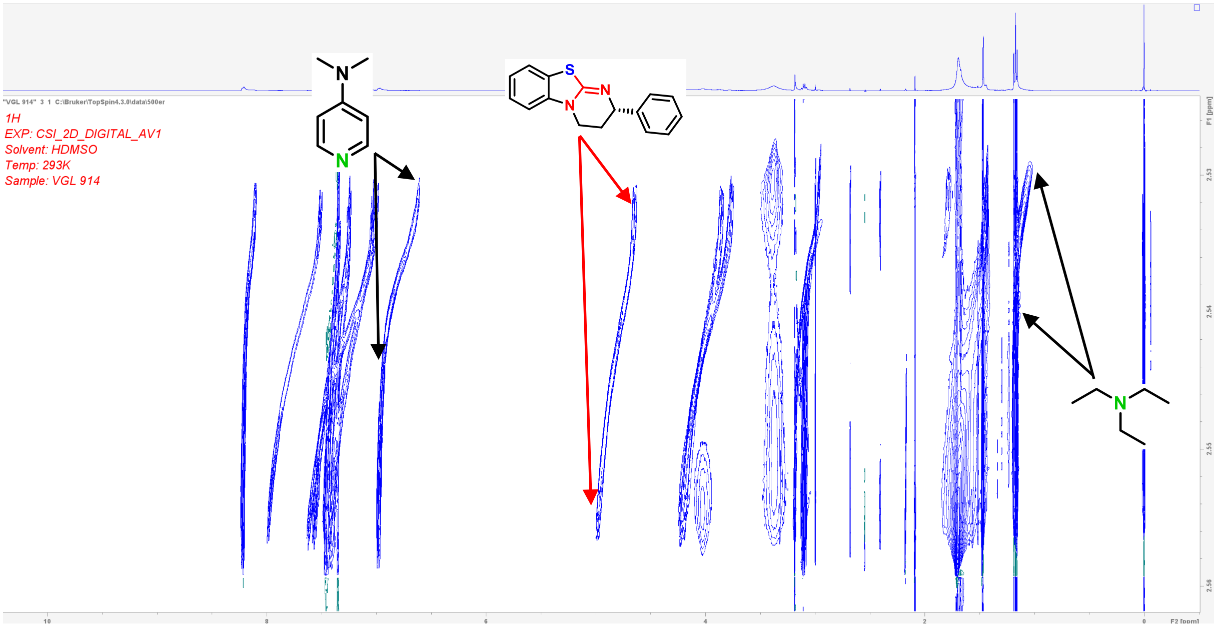

Figure S35: CSI experiment for HBTM in DMSO.

7.2.8. HyperBTM:

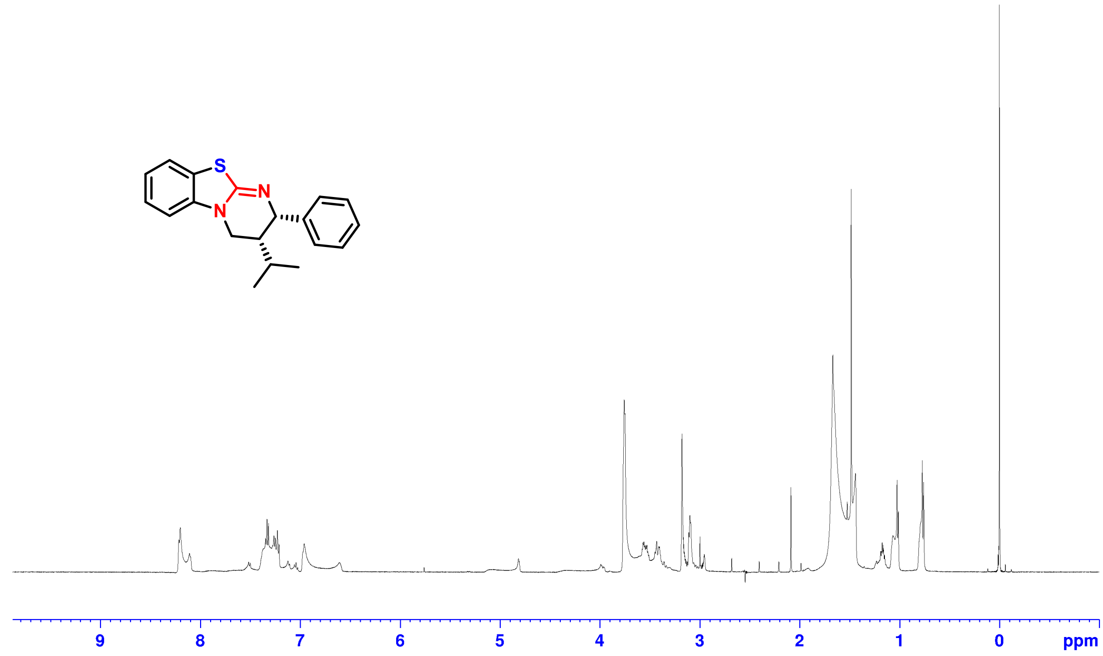

Figure S36: [1]H experiment over whole sample of CSI experiment of HyperBTM in DMSO.

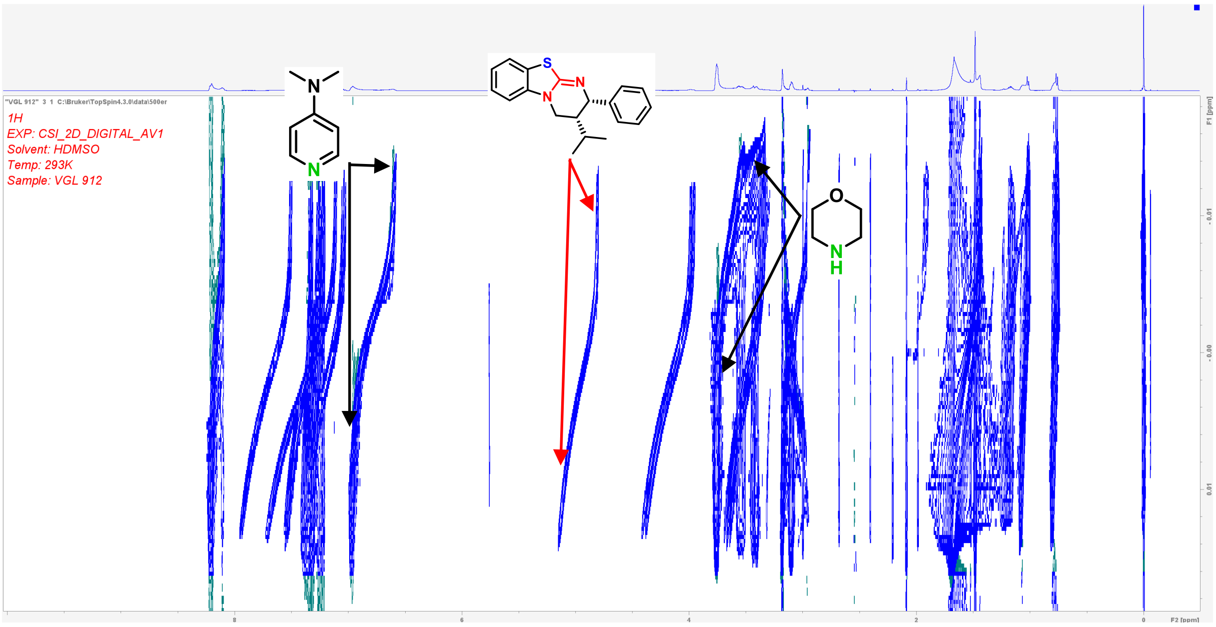

Figure S37: CSI experiment for HyperBTM in DMSO.

7.2.9. DHBP

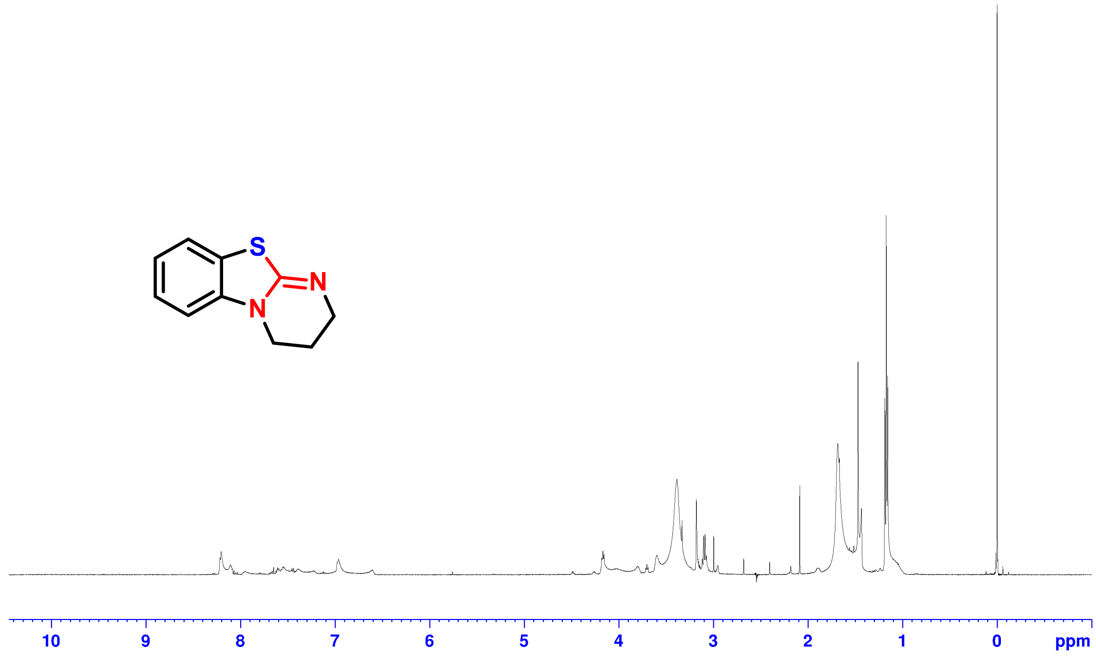

Figure S38: [1]H experiment over whole sample of CSI experiment of DHBP in DMSO.

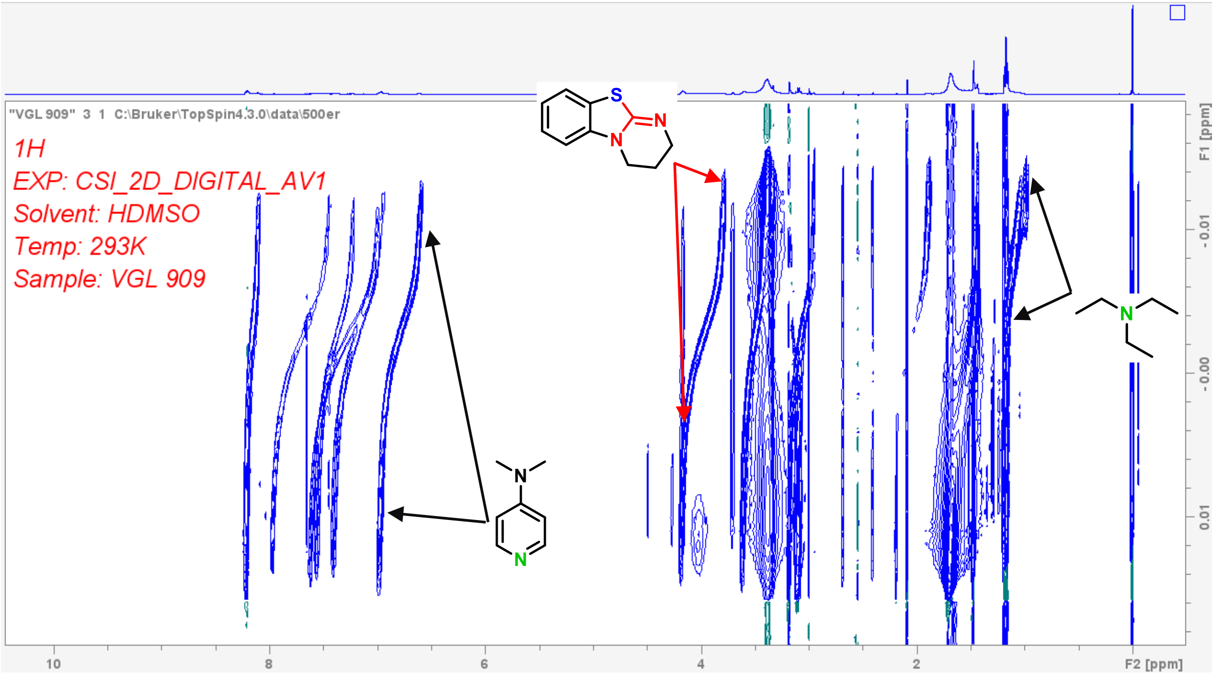

Figure S39: CSI experiment for DHBP in DMSO.
